# Development of an EAT-Lancet index and its relation to mortality in a Swedish population

**DOI:** 10.1093/ajcn/nqab369

**Published:** 2021-11-13

**Authors:** Anna Stubbendorff, Emily Sonestedt, Stina Ramne, Isabel Drake, Elinor Hallström, Ulrika Ericson

**Affiliations:** Department of Clinical Sciences Malmö, Lund University, Malmö, Sweden; Department of Clinical Sciences Malmö, Lund University, Malmö, Sweden; Department of Clinical Sciences Malmö, Lund University, Malmö, Sweden; Department of Clinical Sciences Malmö, Lund University, Malmö, Sweden; Department of Agriculture and Food, Research Institutes of Sweden (RISE), Lund, Sweden; Department of Clinical Sciences Malmö, Lund University, Malmö, Sweden

**Keywords:** sustainable development, environment, diet, nutrition, healthy eating, mortality, survival

## Abstract

**Background:**

Current global food systems threaten human health and environmental sustainability. In 2019, the EAT-Lancet Commission on healthy diets from sustainable food systems defined the first global reference diet to improve both areas, but there is no consensus on how to quantify the EAT-Lancet reference diet as a diet index, and its relation to mortality has not been widely studied.

**Objectives:**

We sought to develop a new dietary index to quantify adherence to the EAT-Lancet diet and assess its association with mortality in a large, population-based Swedish cohort. We also examined food components included in the index and their individual associations with mortality.

**Methods:**

We used the Malmö Diet and Cancer cohort (*n* = 22,421; 45–73 years old at baseline). Dietary data were collected using a modified diet history method. The EAT-Lancet index was developed based on intake levels and reference intervals of 14 food components defined in the EAT-Lancet diet (0–3 points per component; 0–42 points in total). Associations with mortality were examined based on registers during a mean of 20 years of follow-up and were adjusted for potential confounders.

**Results:**

Divided into 5 adherence groups, the highest adherence to the EAT-Lancet diet (≥23 points) was associated with lower all-cause mortality (HR, 0.75; 95% CI, 0.67–0.85), cancer mortality (HR, 0.76; 95% CI, 0.63–0.92), and cardiovascular mortality (HR, 0.68; 95% CI, 0.54–0.84) than the lowest adherence (≤13 points). Several food components included in the index contributed to the observed reductions in mortality.

**Conclusions:**

We developed a new dietary index to investigate adherence to the EAT-Lancet diet. The findings indicate a 25% lower risk of mortality among those with the highest adherence to the EAT-Lancet diet, as defined using our index, which adds to the evidence base for the development of sustainable dietary guidelines.

## Introduction

Shifting to sustainable diets is necessary for achieving the United Nations’ Sustainable Development Goals and maintaining our existence within environmental limits (1–3). The agricultural sector is estimated to be responsible for 25%–30% of the world's greenhouse gas emissions and has a major impact on several other environmental factors (4, 5). Moreover, diet is identified as a major contributor to disease and death globally (6). Noncommunicable diseases account for 71% of all deaths globally, and data indicate that adaption to healthy dietary patterns would substantially reduce the risks of morbidity and mortality from noncommunicable diseases (6–8).

In 2019, the EAT-Lancet Commission on healthy diets from sustainable food systems described the “Great Food Transformation” as a crucial process to keep the impact of food systems within a safe operating space for the planet while feeding the growing global population with healthy diets (9). The commission presented a healthy reference diet (the EAT-Lancet diet; **Supplemental Table 1**), with the aim of both being environmentally sustainable and preventing diet-related chronic diseases and mortality (9). The EAT-Lancet diet is mainly plant based, with a limited content of animal-based foods, sugar, and saturated fat, and with emphasized content of whole grains, vegetables, fruit, legumes, and nuts. The authors estimated that the diet would prevent approximately 11 million deaths per year, equivalent to 19%–24% of total deaths globally, based on projections from aggregated data.

Although the health aspects of the EAT-Lancet diet were based on the literature, knowledge about the health effects of different populations following the EAT-Lancet diet is sparse and has only been evaluated in the context of health outcomes to a limited extent. Some attempts have been made to develop diet scores measuring adherence to the EAT-Lancet diet (10–12). However, there is no consensus as yet on how to quantify the diet, and proposed methods vary depending on the scoring method used and the interpretation of foods to emphasize and limit. For example, previous diet scores have limited the possibility to capture intake variation outside proposed reference levels of the EAT-Lancet diet, as they are comprised of binary food components.

In this study, the aim was to develop a new index to measure adherence to the EAT-Lancet diet and evaluate adherence to the EAT-Lancet diet and its association with mortality in a large, population-based Swedish cohort. We also examined food components included in the index and their individual associations with mortality. The new index is based on a novel scoring system that allows us to measure the degree of adherence to the proposed EAT-Lancet diet reference levels for different foods, and thereby extends the ability to differentiate between individuals’ dietary patterns and associated sustainability performance.

## Methods

### Study design and subjects

The Malmö Diet and Cancer Study (MDCS) is a population-based cohort study that was conducted in the city of Malmö, Sweden, with baseline examinations conducted between 1991 and 1996. Entire birth cohorts of men aged 46–73 years and women aged 45–73 years were recruited for baseline examinations. Recruitment was performed using both community-directed (passive) invitations—that is, advertisements in public areas—and personal letters of invitation (active recruitment). The exclusion criteria were limited Swedish language skills and mental disability that prevented participants from filling out the baseline questionnaire (68,905 eligible). The recruitment process of the MDCS has been described in full detail elsewhere (13, 14).

During the participants’ first visit, anthropometrics and blood pressure were measured and blood samples (nonfasting) were drawn (15). A self-administered questionnaire on lifestyle and socioeconomic factors was completed. The participants received information about how to fill out a food diary and an FFQ. During the second visit 2 weeks later, a diet interview was conducted and their questionnaires were checked (13, 15). In total, 28,098 subjects completed the baseline examinations (40% of the eligible individuals). The MDCS participants were compared with participants in a mailed health survey in Malmö with a higher participation rate (75%). They were found to be similar to nonparticipants regarding sociodemographic factors and lifestyle but to report somewhat better subjective health (13).

The exclusion criteria in this specific study were incomplete information about consumption of any of the studied food groups (*n* = 2128) and missing data on physical activity (*n* = 132), alcohol consumption (*n* = 25), BMI (*n* = 41), smoking habits (*n* = 9), or level of education (*n* = 66). Participants with prevalent diabetes at baseline (*n* = 1131), cardiovascular diseases (*n* = 761), and/or cancer at baseline (*n* = 1627) were also excluded, resulting in a total study population of 22,421 (8568 men and 13,853 women; **Supplemental Figure 1**). The ethics committee at Lund University approved the study (LU 51–90), and the participants provided written informed consent.

### Dietary assessment

Dietary intake was assessed using a validated, modified diet history method consisting of 3 parts: *1*) a 7-day (consecutive days) food diary covering meals that vary from day to day (primarily lunch and dinner), cold beverages (including alcoholic beverages), and dietary supplements; *2*) a 168-item FFQ covering consumption frequencies and portion sizes of food regularly consumed, such as breakfasts and snacks, in the past 12 months and not covered by the food diary; and *3*) a 60-minute interview conducted to ask for cooking methods and usual portion sizes and to check for overlap between intakes reported by the 7-day food diary and the FFQ. The modified diet history method originally developed for the MDCS was found to have good validity and reproducibility; Pearson's correlation coefficients for absolute intake of food groups with reference values in the EAT-Lancet diet ranged in men from 0.35 for fish to 0.82 for meat and in women from 0.58 for vegetables to 0.91 for meat (16). As a result of altered coding routines for dietary data, the interview was shortened to 45 minutes in 1994. The assessed energy intake was slightly lower after the change, but the adjustment had no major impact on the ranking of the participants (17).

The average total food intake (g/day) was summarized from the food diary and the FFQ. Extreme values of total energy, nutrients, major food groups, and portion sizes were checked for errors. Energy and nutrient intakes were calculated using a food composition database (PC-KOST2–93) from the Swedish National Food Agency (1600 food items). Additional recipes and food items were added specifically for this cohort. Total intake of protein, fat, and carbohydrates was expressed as a percentage of the nonalcoholic energy intake (E%). Calculation of added sugar was performed by summing the intakes of sucrose and monosaccharides, followed by exclusion of sucrose and monosaccharides from fruits and vegetables (including juices) (18). The food components included in this study were constructed based on available data regarding the intakes of different food components in the MDCS food database (**Supplemental Table 2**).

### EAT-Lancet index

We developed an index to validate the EAT-Lancet diet regarding its association with mortality. The EAT-Lancet diet consists of food components for which defined target intake levels and reference intervals (ranges) are suggested (Supplemental Table 1). The reference intervals are described as uncertainty ranges and are considered to be compatible with optimal health in different populations (9). In this study, food components were classified as either “emphasized foods” or “limited foods” based on previous descriptions of the EAT-Lancet diet (9, 19). Emphasized food components were vegetables, fruits, unsaturated oils, legumes, whole grains, nuts, and fish. Food components classified as limited were beef and lamb, pork, poultry, eggs, dairy, potatoes, and added sugar. Dietary intakes were evaluated based on reported amounts in grams per day in uncooked weight, which is in line with how the target intake levels are expressed in the EAT-Lancet diet (see details of included food components in Supplemental Table 2). Our index consists of the 14 food components, with a possible range of 0–3 points for each component; 0 points indicates low adherence to the target for the food component in the EAT-Lancet diet and 3 points indicates high adherence, as described in **Table 1**. The total possible score range of the developed index is 0 (nonadherence) to 42 points (perfect adherence = 14 × 3 points).

**TABLE 1:** Criteria for the EAT-Lancet index constructed to evaluate the EAT-Lancet diet in relation to mortality in the Malmö Diet and Cancer Study

Food components in the EAT-Lancet diet index^1^		Target intake (reference interval)^2^	3 points	2 points	1 point	0 points	Criteria for score distribution
Emphasized intake	Vegetables	300 (200–600)	>300	200–300	100–200	<100	Positive score 3 points = intake above target intake 2 points = lower limit of reference interval up to target intake 1 point = 50%–100% of lower limit of reference interval 0 points = <50% of lower limit of reference interval
	Fruits	200 (100–300)	>200	100–200	50–100	<50	
	Unsaturated oils	40 (20–80)	>40	20–40	10–20	<10	
	Legumes	75 (0–150)	>75	37.5–75	18.75–37.5	<18.75	Positive score, adjusted^3^ 3 points: intake above target intake 2 points: 50%–100% of target intake 1 point: 25%–50% target intake 0 points: 0%–25% of target intake
	Nuts	50 (0–100)	>50	25–50	12.5–25	<12.5	
	Whole grains	232	>232	116–232	58–116	<58	
	Fish	28 (0–100)	>28	14–28	7–14	<7	
Limited intake	Beef and lamb	7 (0–14)	<7	7–14	14–28	>28	Inverse score 3 points: intake below target intake 2 points: target intake to upper limit of reference interval 1 point: 100%–200% of upper limit of reference interval 0 points: >200% of upper limit of reference interval
	Pork	7 (0–14)	<7	7–14	14–28	>28	
	Poultry	29 (0–58)	<29	29–58	58–116	>116	
	Eggs	13 (0–25)	<13	13–25	25–50	>50	
	Dairy	250 (0–500)	<250	250–500	500–1000	>1000	
	Potatoes	50 (0–100)	<50	50–100	100–200	>200	
	Added sugar^4^	31 (0–31)	<31	31–62	62–124	>124	

1 Food components in the index are based on the EAT-Lancet diet as grams per day, with some modifications. Vegetables are described as a single group since no information about subgroups (i.e., green or red vegetables) was available in the Malmö Diet and Cancer Study. Fat intake and quality are reflected as unsaturated oils and plant margarines, since no information about palm oil or lard was available.

2 Target and reference values from the EAT-Lancet diet, based on an energy intake of 2500 kcal, expressed in grams (9).

3 Initial criteria for the positive score were not applicable, as the lower limit of the reference interval was set to 0 for those foods.

4 Since the upper limits of the reference interval and target were identical, we used an upper reference interval of target intake × 2 (62 g). An upper limit of the reference interval of 62 g for added sugar is in line with the WHO recommendation of ≤10% of the nonalcoholic energy intake (20).

### Assessment of covariates

The participants’ weight and height were measured, and BMI was calculated as kg/m^2^. Weight was classified as normal (BMI < 25 kg/m^2^), overweight (BMI 25–29 kg/m^2^) and obese (BMI > 30 kg/m^2^). A questionnaire was used at baseline to assess the participants’ demographic, lifestyle, socioeconomic, and social factors; medical history; and use of medical and dietary supplements. Leisure-time physical activity was assessed as time spent per week on 17 different activities. These figures per activity were multiplied by their respective intensity factor. This was added to a total and the participants were divided into quintiles of leisure-time physical activity (21). Alcohol consumption was defined by a 4-category variable. Participants reporting 0 consumption in the 7-day food diary and indicating no consumption during the previous year in the lifestyle questionnaire were categorized as 0 reporters. The other categories were <15 g alcohol/day for women and <20 g/day for men (low), 15–30 g/day for women and 20–40 g/day for men (medium), and >30 g/day for women and >40 g/day for men (high), based on the recorded consumption in the food diary. Smoking habits were divided into 3 categories of reported habits (smokers, former smokers, and never smokers at baseline). Education level was divided into 4 categories according to the participants’ highest level of education (≤8 years, 9–10 years, 11–13 years, or university degree). Season was divided into 4 categories: winter (January–March), spring (April–June), summer (July–September), and fall (October–December). Due to a minor adjustment in coding routines of dietary data in September 1994, which shortened the dietary interview from 60 minutes to 45 minutes, the dietary assessment version was introduced as a variable (old and new). Participants who reported substantial dietary changes in the past during the baseline examination were classified as past diet changers. Potential under- and overreporters of energy were defined according to Black and Goldberg (22) using individual values for physical activity level; that is, participants having a ratio of the reported energy intake to the basal metabolic rate outside the 95% CI of their calculated physical activity level. This method has been described more extensively elsewhere (23).

### Assessment of mortality

Vital status and emigration status were obtained from the Swedish National Tax Agency, Statistics in Sweden, and the National Board of Health and Welfare until 31 December 2016. The Swedish Cause of Death Register was used to identify causes of death. Cardiovascular death was identified according to International Classification of Diseases, Ninth Revision (ICD-9), codes 390–459 or ICD, Tenth Revision (ICD-10), code I, and cancer death was identified according to ICD-9 codes 140–239 or ICD-10 code C-D48.

### Statistical analysis

The participants were divided into 5 groups according to their total index score (≤13, 14–16, 17–19, 20–22, and ≥23 points). This was based on the aims of making the groups as similar as possible in size while maintaining the same score interval in the groups and of avoiding making groups so small that the extreme groups might include many outliers with unreliable dietary data. The lowest-scoring group, with ≤13 points, was defined as having low adherence, and the highest scoring group, with ≥23 points, was defined as having high adherence. In a post hoc analysis, 7 groups were used instead of 5.

The differences in characteristics of participants in the 5 groups were tested using a chi-square test for categorical variables and using a general linear model for continuous variables. For the cross-sectional analysis of the participants’ characteristics measured at baseline, the means, 95% CIs, and tests for trends were adjusted for sex, age, season, and dietary assessment version.

Cox proportional hazards regression was used to examine the associations between the EAT-Lancet score and HRs of all-cause mortality, cancer mortality, and cardiovascular mortality. The group with the lowest adherence to the EAT-Lancet diet (≤13 points) was used as the reference group, and years of follow-up was used as the underlying time variable. Model 1 (basic model) was adjusted for age, sex, dietary assessment version, season, and energy intake; Model 2 was adjusted for the covariates included in model 1 plus leisure-time physical activity, smoking habits, alcohol consumption, and educational level; Model 3 was adjusted for the covariates included in Model 2 plus BMI; and Model 4 was adjusted for age, sex, dietary assessment version, season, physical activity, smoking habits, alcohol consumption, and education level (i.e., no adjustment for energy intake and BMI). The covariates included in the model were identified from the literature to indicate potential confounding of diet-disease associations. One reason for excluding energy and BMI in Model 4 was that associations potentially mediated by energy intake and BMI also are of interest. The fully adjusted model (Model 3) was used as the main model, and the results below are based on this model unless otherwise stated. We also examined the index as a continuous variable (0–42 points) in relation to all-cause mortality.

We used a Cox proportional hazards model and the “*survival*” package in R software, version 4.0.2 (R Project for Statistical Computing), to estimate the standardized 10-, 15-, and 20-year absolute risks of mortality by the EAT Lancet-index. Absolute risk estimates were standardized to the mean of all covariates in Model 3. To assess the proportional hazards assumption, we tested interactions between the underlying time variable and examined covariates with regard to all-cause and cause-specific mortality. The assumption was not considered to be satisfied for the covariates of age, dietary assessment version, and smoking (*P* values for interaction < 0.05). We therefore additionally performed mortality analyses with stratified Cox models (per 1-year age interval, dietary assessment version, and smoking), but our reported results remained unchanged. Reported intake of all 14 food groups was also tested individually regarding their associations with all-cause mortality, cancer mortality, and cardiovascular mortality. For those analyses, we also added an additional model (Model 5) including all food components simultaneously. We also performed a sex-specific analysis, as it is well known that dietary habits differ between women and men and that they tend to misreport dietary intake to different extents (24, 25). A sensitivity analysis was performed excluding energy misreporters, participants reporting dietary changes, and participants who died within 2 years after the baseline examinations.

The dose-response relationships between the 14 food groups included in the EAT-Lancet index and all-cause mortality were studied using restricted cubic splines of the Cox regression Model 3. The reference was placed at 0 g/day for the respective food groups, and the lowest observed score (5 points) for the EAT-Lancet index was used as the reference. Four knots were placed at Harrell's (26) default quantiles. However, due to the large number of 0 consumers of poultry, legumes, and nuts, these food components were instead studied with 3 knots; Harrell's (26) default quantiles were used for poultry and legumes, and the knots were placed at 10, 20, and 30 g/day for nuts to allow fitting of the spline.

The statistical analyses were performed using SPSS (IBM SPSS Statistics for Windows, Version 27.0; IBM Corp), Stata/SE (version 15.0; StataCorp LLC), and R software, version 4.0.2 (R Project for Statistical Computing). A 2-tailed *P* value < 0.05 was considered significant.

## Results

### Baseline characteristics

**FIGURE 1:**
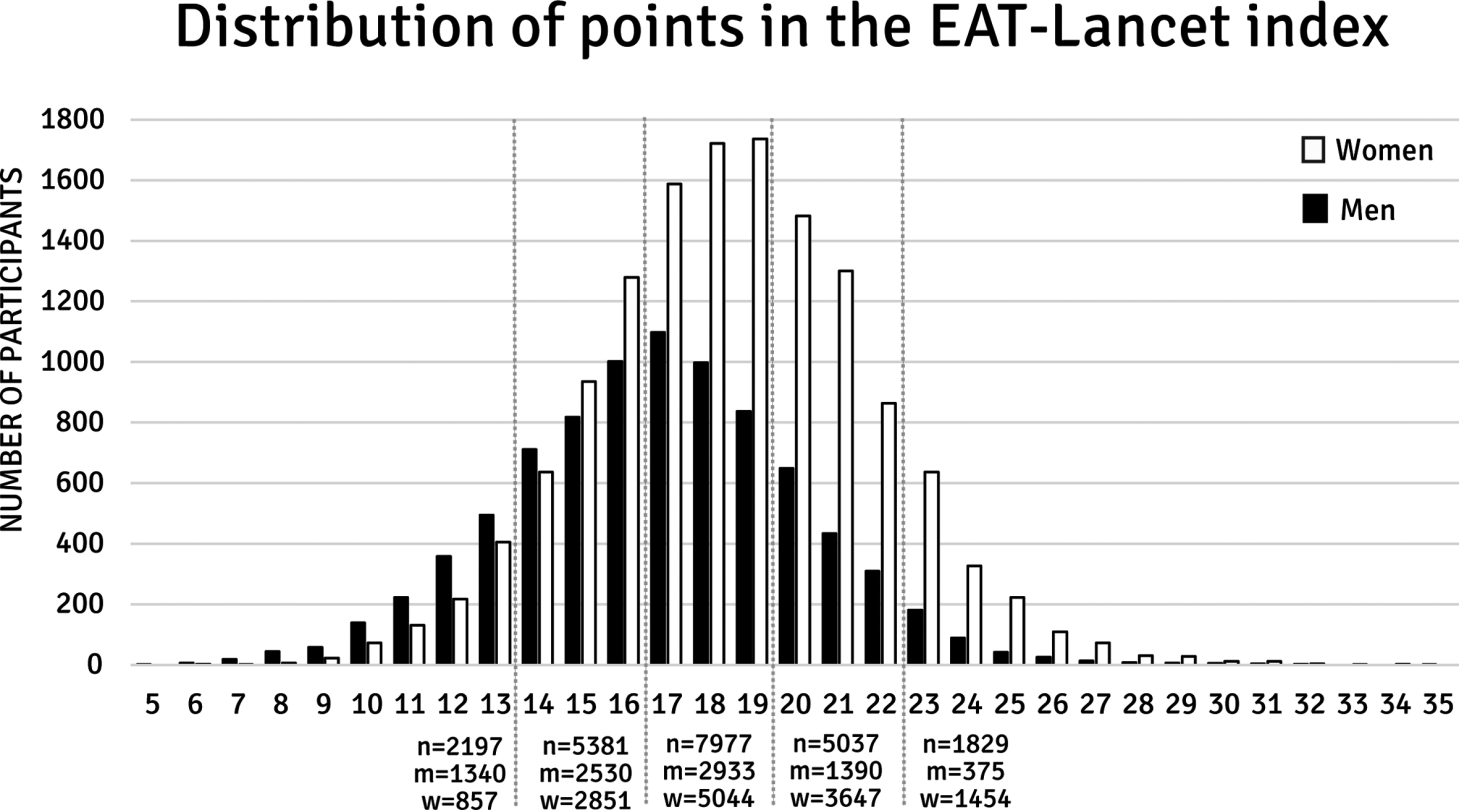
Distribution of points among 8,568 men (m) and 13,853 women (w) in the Malmö Diet and Cancer Study (possible range, 0–42 points). The population was divided into 5 categories in the analyses (≤13, 14–16, 17–19, 20–22, ≥23), and *n* indicates the number of participants in each group.

The results showed large variations in adherence to the EAT-Lancet diet among individuals and among specific food groups. Participants in the MDCS obtained between 5 and 35 points on the EAT-Lancet index (range, 0–42 points), with a mean of 17.9 points (SD, ±3.4 points). Women had higher adherence to the EAT-Lancet diet, with a mean score of 18.5 (SD, ±3.3), while the mean score for men was 16.8 (SD, ±3.4; **Figure 1**).

**FIGURE 2:**
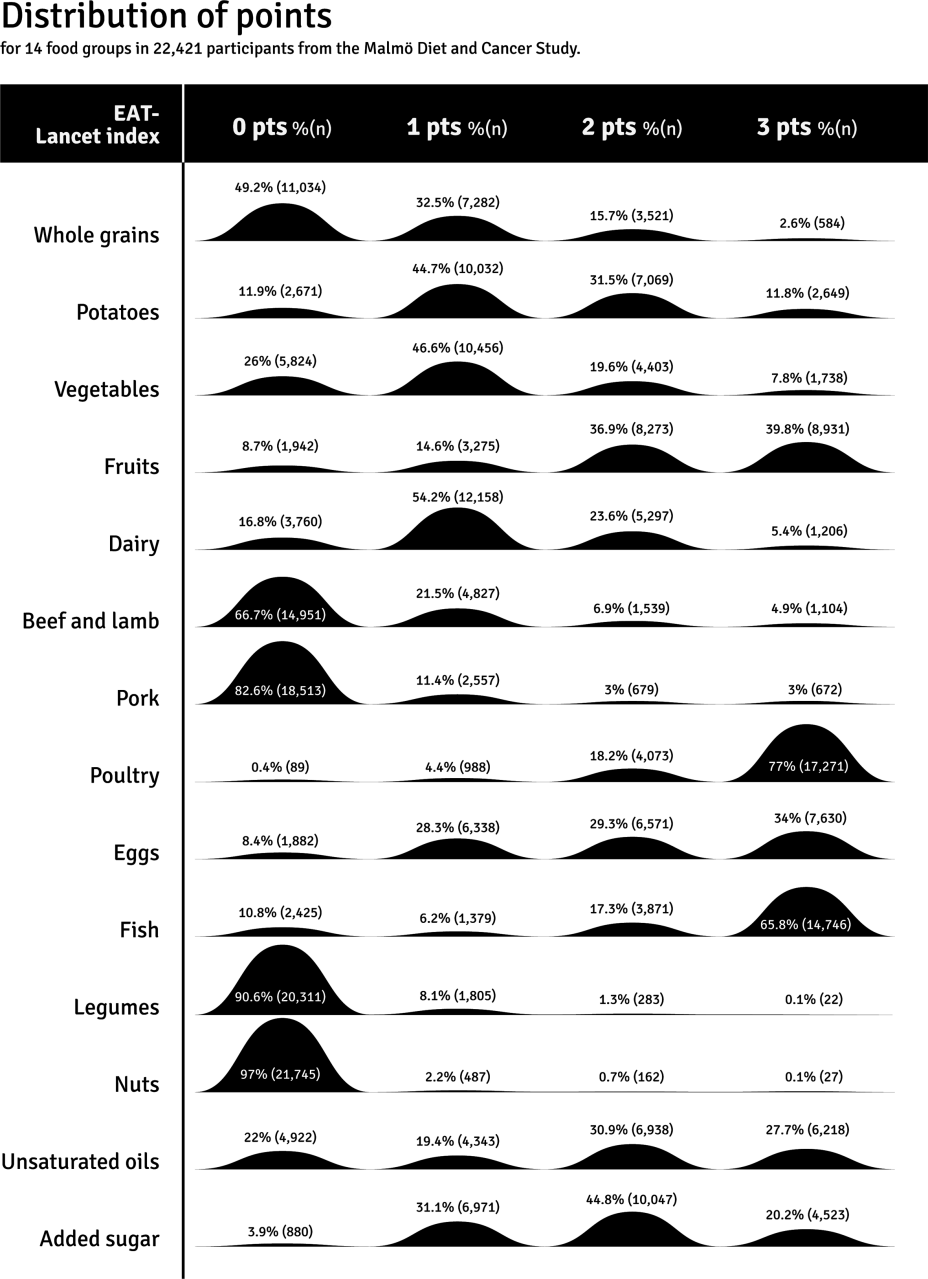
Distribution of EAT-Lancet index points for 14 food groups in 22,421 participants from the Malmö Diet and Cancer Study. A very low percentage (<5%) of the participants reached the target intakes defined in the EAT-Lancet diet (3 points) for legumes, nuts, whole grains, pork, and beef and lamb, whereas most of the participants reached the target intakes for poultry and fish (<65%).

**FIGURE 3:**
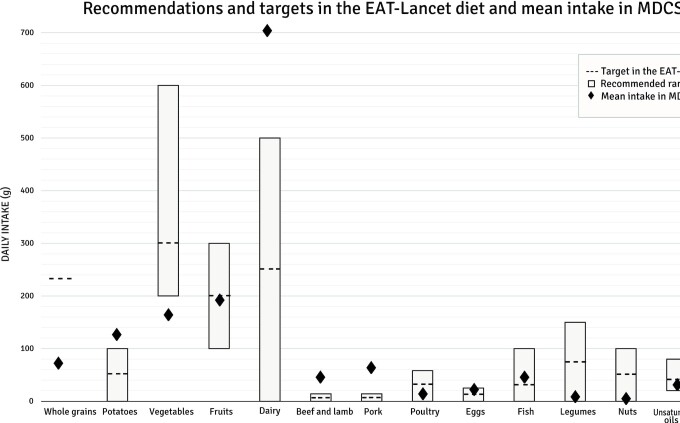
Recommended range and targets in the EAT-Lancet diet, compared with the average intake among 22,421 participants in the MDCS. Abbreviation: MDCS, Malmö Diet and Cancer Study.

Less than 1% of the study population reached the target intake of the EAT-Lancet diet for legumes and nuts. Additionally, less than 5% reached the target intakes for whole grains, beef and lamb, and pork. Adherence was highest for poultry and fish, where 77% and 66% of the population reached the target intakes, respectively (**Figure 2**). The mean absolute intakes compared to the target and reference intervals in the EAT-Lancet diet are shown in **Figure 3**. Women had higher index points regarding all food components except for whole grains, fish, and unsaturated oils (**Supplemental Table 3**). Low-to-moderate correlations were observed between intakes of different food components included in the EAT-Lancet index (**Supplemental Table 4**). The highest correlations (r > 0.3) were observed between fruits and vegetables, between potatoes and beef and lamb, and between potatoes and pork.

Those with the high adherence to the EAT-Lancet diet (higher score) had lower total energy intake and a diet containing lower E% from fat, higher E% from carbohydrates, more dietary fiber, and a marginally higher E% from protein compared to those with low adherence (**Table 2**). The participants with high adherence to the EAT-Lancet diet were slightly older and less often smokers, and fewer among them had high alcohol consumption. In addition, university degrees and high leisure-time physical activity were more common in this group. BMI did not differ across categories of the EAT-Lancet index in the analysis of women and men together, but in men, BMI increased slightly with higher adherence to the EAT-Lancet diet, and this trend was also reflected by a lower percentage of normal-weight men in the group with higher adherence (**Supplemental Table 5**).

**TABLE 2:** Participant characteristics and EAT-Lancet score in 22,424 participants from the Malmö Diet and Cancer Study^1^

	EAT-Lancet index categories				
	≤13	14–16	17–19	20–22	≥23
*n*	2197	5381	7977	5037	1829
Age,^2^ years	56.15 (55.83–56.47)	57.24 (57.03–57.44)	57.97 (57.81–58.14)	58.32 (58.11–58.53)	57.86 (57.51–58.21)
BMI,^3^ kg/m^2^	25.26 (25.10–25.42)	25.55 (25.45–25.65)	25.78 (25.69–25.86)	25.68 (25.58–25.79)	25.20 (25.02–25.38)
Energy intake,^4^ kcal	2530 (2507–2554)	2374 (2359–2389)	2257 (2245–2270)	2164 (2148–2179)	2108 (2083–2134)
Fat,^4^ E%	41.98 (41.73–42.23)	39.89 (39.73–40.04)	38.33 (38.20–38.45)	36.58 (36.41–36.74)	35.07 (34.80–35.34)
Carbohydrates,^4^ E%	43.29 (43.04–43.54)	44.89 (44.73–45.05)	46.13 (46.00–46.26)	47.71 (47.54–47.87)	49.53 (49.25–49.80)
Protein,^4^ E%	14.73 (14.63–14.84)	15.22 (15.16–15.29)	15.55 (15.50–15.60)	15.72 (15.65–15.78)	15.41 (15.30–15.52)
Dietary fiber,^4^ g	15.77 (15.50–16.03)	17.82 (17.66–17.99)	19.75 (19.61–19.88)	21.85 (21.67–22.02)	25.34 (25.05–25.63)
Dietary fiber,^4^ g/MJ	1.54 (1.52–1.56)	1.82 (1.80–1.83)	2.11 (2.10–2.12)	2.45 (2.44–2.46)	2.95 (2.92–2.97)
Overweight: BMI 25–29.99 kg/m^2^	40.6%	39.6%	40.2%	38.9%	34.2%
Obese: BMI > 30 kg/m^2^	10.7%	12.2%	13.3%	12.1%	10.6%
BMI < 25 kg/m^2^	48.70%	48.20%	46.50%	49.00%	55.20%
Current smoker	47.3%	35.5%	25.8%	21.3%	20.5%
High alcohol intake^5^	7.1%	5.8%	4.0%	3.0%	3.4%
University degree	10.0%	13.3%	14.8%	17.0%	20.3%
High physical activity^6^	14.7%	18.1%	20.0%	22.7%	26.4%

1 Values are means with 95% CI or percentages. E%, percentage of the nonalcoholic energy intake.

2 Adjusted for sex.

3 Adjusted for age and sex.

4 Adjusted for age, sex, dietary assessment version, and season.

5 High alcohol consumption is defined as above 30 g/d for women and above 40 g/d for men.

6 Highest leisure time physical activity quintile.

### The EAT-Lancet index and mortality

**FIGURE 4:**
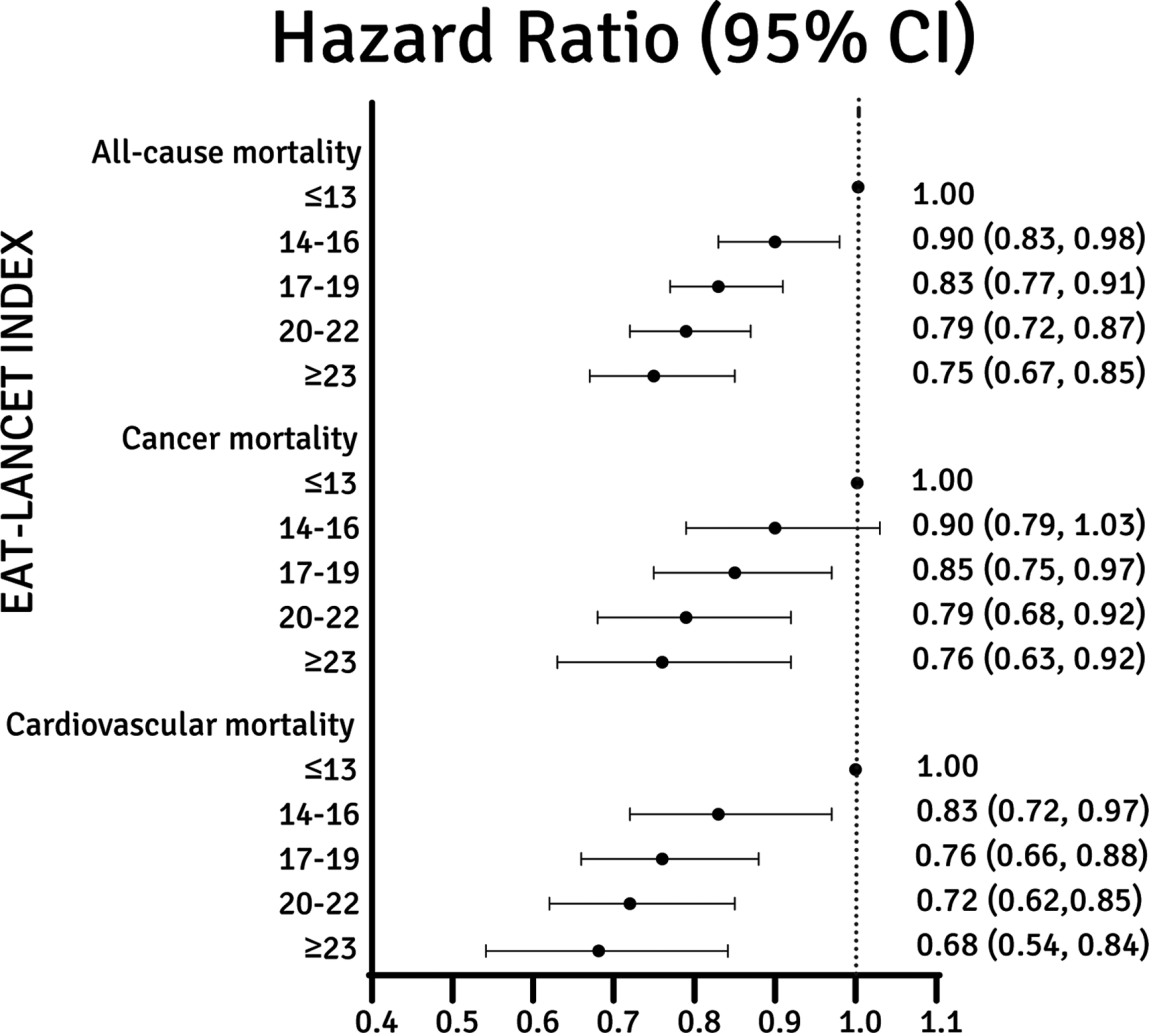
EAT-Lancet score and HR with 95% CI in 22,421 participants in Malmö Diet and Cancer Study, based on the fully adjusted model (Model 3). High adherence to the EAT-Lancet diet (≥23 points) was associated with 25% lower all-cause mortality, 24% lower cancer mortality, and 32% lower cardiovascular mortality than low adherence (≤13 points).

During a mean of 20 years of follow-up, 7030 deaths occurred (31.4%). In the fully adjusted model, the highest adherence to the EAT-Lancet diet (≥ 23 points) was associated with a 25% lower risk of all-cause mortality (HR, 0.75; 95% CI, 0.67–0.85; *P* trend < 0.001) compared to low adherence (≤13 points; **Figure 4**; **Supplemental Figure 2; Supplemental Table 6**).

The estimated HRs across categories of the EAT-Lancet index corresponded to a 10-year absolute risk of mortality of 3.7% (95% CI, 3.3%–4.1%) in the group with the highest adherence and 5.1% (95% CI, 4.7%–5.5%) in the group with the lowest adherence (**Supplemental Table 7**). Similar associations were seen among women and men (**Supplemental Tables 8**and **9**). All-cause mortality Kaplan-Meier plots are shown in **Supplemental Figures 3–5**. When evaluating the EAT-Lancet index as a continuous variable (0–42 points), each incremental increase in points resulted in a 4% lower risk of all-cause mortality (HR, 0.956: 95% CI, 0.949–0.964). Moreover, both cancer and cardiovascular mortality risks were significantly lower [HRs, 0.76 (95% CI, 0.63–0.92) and 0.68 (95% CI, 0.54–0.84), respectively] when comparing the highest adherence group to the lowest (Supplemental Table 6). Sex-specific analyses showed a lower risk of cardiovascular and cancer mortality in both sexes when comparing the highest adherence group to the lowest (Supplemental Tables 8 and 9). When assessing the model without adjustments for BMI or energy intake (Model 4), only minor differences were seen compared to Model 3. To analyze those with exceptionally poor or good adherence to the EAT-Lancet diet, post hoc analyses were performed in which the participants were divided into 7 adherence groups instead of 5. In these analyses, participants with the highest adherence (≥26 points) had a 39% lower risk of all-cause mortality than those with the lowest adherence (≤10 points; HR, 0.61; 95% CI, 0.48–0.77; **Supplemental Table 10**).

The observed inverse association between the EAT-Lancet index and all-cause mortality remained virtually unchanged in sensitivity analyses, excluding participants classified as potential under- or overreporters of energy (*n* = 4022), participants reporting a dietary change (*n* = 4643), and participants who died less than 2 years after baseline examinations (*n* = 152; **Supplemental Table 11**).

Adherence to each of the 14 food group components included in the EAT-Lancet index and mortality were also tested separately (**Table 3**; **Supplemental Tables 12–14**; Supplemental Figure 2). High intakes of whole grains, vegetables, and fruits, compared to low intakes, were associated with lower risks of all-cause mortality, whereas high intake of eggs was associated with a higher risk. In addition, high intake of potatoes tended to be associated with a higher risk of mortality than did low intake, but this tendency was attenuated after mutual adjustment of all food components. Similar associations were observed between the food components and cause-specific mortality: that is, cancer mortality and cardiovascular mortality.

**TABLE 3:** Associations between EAT-Lancet index components (14 food groups) and all-cause mortality in 22,424 participants from the Malmö Diet and Cancer Study stratified according to their score^1^

	EAT-Lancet index component points				
	0	1	2	3	*P* trend^2^
Whole grains (*n*)	<58 g (11,034)	58–116 g (7282)	116–232 g (3521)	>232 g (584)	
Model 3^3^	1	0.91 (0.86–0.96)	0.92 (0.86–0.99)	0.81 (0.70–0.95)	<0.001
Potatoes (*n*)	>200 g (2671)	100–200 g (10,032)	50–100 g (7069)	<50 g (2649)	
Model 3^3^	1	0.92 (0.86–0.99)	0.91 (0.83–0.98)	0.90 (0.81–1.00)	0.05
Vegetables (*n*)	<100 g (5824)	100–200 g (10,456)	200–300 g (4403)	>300 g (1738)	
Model 3^3^	1	0.90 (0.86–0.95)	0.85 (0.79–0.91)	0.76 (0.68–0.85)	<0.001
Fruits (*n*)	<50 g (1942)	50-100 g (3275)	100-200 g (8273)	>200 g (8931)	
Model 3^3^	1	0.87 (0.79–0.96)	0.85 (0.78–0.93)	0.80 (0.73–0.88)	<0.001
Dairy (*n*)	>1000 g (3760)	500–1000 g (12,158)	250–500 g (5297)	<250 g (1206)	
Model 3^3^	1	0.93 (0.87–1.00)	0.94 (0.86–1.02)	1.04 (0.92–1.17)	0.98
Beef and lamb (*n*)	>28 g (14,951)	14–28 g (4827)	7–14 g (1539)	<7 g (1104)	
Model 3^3^	1	0.96 (0.90–1.02)	0.95 (0.87–1.05)	0.92 (0.82–1.04)	0.06
Pork (*n*)	>28 g (18,513)	14–28 g (2557)	7–14 g (679)	<7 g (672)	
Model 3^3^	1	0.99 (0.91–1.07)	0.93 (0.81–1.08)	0.91 (0.78–1.07)	0.78
Poultry (*n*)	>116 g (89)	58–116 g (988)	29–58 g (4073)	<29 g (17,271)	
Model 3^3^	1	0.75 (0.50–1.11)	0.80 (0.55–1.18)	0.80 (0.55–1.17)	0.63
Eggs (*n*)	>50 g (1882)	25–50 g (6338)	13–25 g (6571)	<13 g (7630)	
Model 3^3^	1	0.92 (0.84–1.00)	0.86 (0.79–0.94)	0.90 (0.82–0.98)	0.03
Fish (*n*)	<7 g (2425)	7–14 g (1379)	14–28 g (3871)	>28 g (14,746)	
Model 3^3^	1	1.05 (0.92–1.19)	1.02 (0.92–1.12)	0.97 (0.89–1.05)	0.14
Legumes (*n*)	<18.75 g (20,311)	18.75–37.5 g (1805)	37.5–75 g (283)	>75 g (22)	
Model 3^3^	1	1.02 (0.94–1.11)	1.24 (1.03–1.49)	1.30 (0.65–2.60)	0.06
Nuts (*n*)	<12.5 g (21,745)	12.5–25 g (487)	25–50 g (162)	>50 g (27)	
Model 3^3^	1	0.96 (0.81–1.14)	0.90 (0.67–1.21)	0.85 (0.40–1.78)	0.36
Unsaturated oils (*n*)	<10 g (4922)	10–20 g (4343)	20–40 g (6938)	>50 g (6218)	
Model 3^3^	1	0.99 (0.92–1.07)	0.94 (0.88–1.00)	0.98 (0.91–1.05)	0.28
Added sugar (*n*)	>124 g (880)	62–124 g (6971)	31–62 g (10,047)	<31 g (4523)	
Model 3^3^	1	0.90 (0.80–1.02)	0.91 (0.80–1.03)	0.97 (0.84–1.12)	0.32

1 Values are given as HRs and 95% CIs within parentheses, with the respective 0-point group as the reference group. *n* indicates the number of participants per index group. Multivariate proportional hazards were used to examine the associations.

2 *P* trends were calculated across index categories.

3 Adjusted for age, sex, dietary assessment version, season, energy intake, physical activity, smoking, alcohol use, education, and BMI.

## Discussion

In this study, we developed an index to quantify adherence to the EAT-Lancet diet and assess its relation to mortality in a large, population-based Swedish cohort. Our main findings showed that those with the highest adherence to the EAT-Lancet diet had a 25% lower risk of mortality, with a clear linear trend. Detailed analyses of food components included in the diet indicated that several food components contributed to this observation, which highlights the importance of an overall healthy diet.

Few studies have examined the associations between the EAT-Lancet diet and mortality. In contrast to our results, Knuppel et al. (10) did not observe an association with all-cause mortality in the European Prospective Investigation into Cancer and Nutrition (EPIC) Oxford cohort (46,069 participants and 4214 deaths). Their index has also been used for the whole EPIC cohort, suggesting that 19%–63% of all deaths could be prevented by adherence to the EAT-Lancet diet (comparing the groups with lowest and highest adherence) (27). Their index differed from ours since it was built on binary assessments of each diet component; thus, the possible range was narrower (0–14 points) and different levels of adherence to the proposed intake ranges could not be examined. In contrast to our index, the index developed by Knuppel et al. (10) defined the food groups of legumes, fish, and whole grains as limited foods. We defined them as emphasized because they were described to have positive health outcomes, and increased consumption was encouraged when the EAT-Lancet reference diet was proposed (9).

In a modeling analysis, Springmann et al. (28) estimated that following a plant-based diet, such as the EAT-Lancet diet, could lead to reductions in premature mortality by 19%–22%. Wang et al. (29) further suggested that the EAT-Lancet diet could prevent 25% of total deaths, corresponding to 11 million premature deaths globally, based on a modeling analysis of diet quality. Energy intake has been stressed as a major factor explaining the proposed reductions in premature mortality due to chronic diseases (30). However, in our study, the significant association between diet and mortality remained after adjustments for energy intake and BMI.

In our study, several of the food components included in the index, including higher consumption of whole grains, vegetables, and fruits and lower consumption of eggs, seemed to contribute to the lower mortality associated with high adherence to the EAT-Lancet diet. Although potatoes were classified as a limited food group, the tendency of an increased risk of mortality to be associated with a high potato intake was somewhat unexpected, because previously observed associations with chronic disease seem to be explained by high intake of fried potatoes (31, 32), and potatoes were mainly consumed boiled within this study population. However, the literature on potato intake and mortality is inconclusive (33–35). Regarding seafood, there might be a potential conflict between dietary intake recommendations and sustainability, depending on the type of fish consumed and the production methods used (36), but based on the previously described rationale, fish was classified as emphasized in our index. All other animal foods were classified as limited in this EAT-Lancet index, but indications of higher mortality were only seen for high intake of eggs, and those findings are in line with those of other studies (37). The mean intake of legumes in our study was only 6 g/d, which is far from the target of 75 g in the EAT-Lancet diet, and only 22 participants (0.1%) received 3 points for this component. This indicates that the intake ranges make the study population unsuitable for examination of health outcomes in relation to legume intakes, even if we have no reason to think that the detailed diet assessment method would have provided less satisfactory estimates of legume intakes compared to other food groups. Other studies have observed large inconsistencies regarding the consumption of legumes in relation to mortality and morbidity (37–39). Consumption of nuts was also negligible in this study and far from proposed targets in the EAT-Lancet diet, which hampers possibilities to capture potential relations to mortality.

The strengths of this study were the extensive follow-up period of 20 years on average. The dietary assessment method used in the Malmö Diet and Cancer cohort measures both habitual and recent intakes, and the documented relative validity and reproducibility of food intakes indicate high-quality dietary data. In addition, we were able to exclude participants with suspected unstable food habits before the baseline visit (16, 40). Nevertheless, it is a limitation that diet was assessed with a single dietary measurement at baseline. However, reproducibility studies including participants of similar age as those in our study have shown acceptable agreement between repeated dietary measurements (41, 42), and we therefore expect relatively stable food habits in this middle-aged and elderly population during follow-up. Another limitation might be that very few participants in our study followed the overall EAT-Lancet dietary recommendations: no participants were close to receiving 42 points in the index, and a potential additional reduction in mortality might have been seen for higher scores. Indeed, our post hoc analysis with more extreme groups pointed in that direction. Moreover, all dietary assessment methods are prone to a certain degree of misreporting, and individuals can only be ranked on different intake levels when comparing them. Consequently, we cannot relate health outcomes to exact intake levels, and our results regarding adherence to the EAT-Lancet diet should only be interpreted as relative adherence, although our index was constructed with absolute intake levels based on the target intakes proposed in the EAT-Lancet diet. Vegetables were divided into categories based on color in the EAT-Lancet diet; this was not possible in our study due to the lack of data on subgroups of vegetables. Furthermore, Willett et al. (9) suggested interchangeability between different food groups (e.g., chicken and other poultry are exchangeable with eggs, fish, or plant proteins) in the EAT-Lancet diet, a dimension we could not include in our index. By using absolute scores (0–3) in the index, relative adherence is partly ignored. However, as we wanted to take both proposed target intakes and reference intervals into account, we think that relative scores would be more difficult to reproduce, which may complicate replication of our findings. In addition, we think that there may be a risk that some of the food components would get more weight with relative scores and that such a design may lead to more subjective decisions when constructing the index. Finally, since our main objective was to examine all-cause mortality, we did not take competing risks into account in our statistical analysis, but we cannot exclude that this may be a concern regarding the results on cause-specific mortality.

In future studies, our index could also be examined in relation to the incidence of chronic diseases. In addition, other associated sustainability benefits require further research (43, 44): for example, by linking our index components to life cycle assessment data to estimate how environmental impact varies depending on adherence to the EAT-Lancet diet. Moreover, although modeling studies show that adequate intake of most nutrients is met with the EAT-Lancet diet (9, 45), the nutritional adequacy of the EAT-Lancet diet as assessed with our index has not been studied and might also be of interest. Applying the index on a diet consumed today would also be an interesting area of future research, since we expect different co-consumption patterns today compared to the dietary habits in the 1990s examined in this cohort. Last, evaluating our index in other populations, including those of low- or middle-income countries, would also be relevant to test and develop it for global usage. Similarly, it would be valuable to examine the previously described EAT-Lancet score by Knuppel et al. (10) in the MDCS cohort and compare the findings.

We are familiar with the debate that has surrounded the EAT-Lancet diet due to methodological considerations, lack of adaptation to local contexts and different populations, and feasibility in terms of costs and missing environmental perspectives (12, 30, 46–49). Nevertheless, the considerable reductions in mortality shown in this study provide strong arguments for prioritizing healthy diets similar to the EAT-Lancet diet in policy, in education, and on the overall global agenda.

In conclusion, we developed a new dietary index to investigate adherence to the EAT-Lancet diet, and this study indicates that the EAT-Lancet diet, as assessed with this EAT-Lancet index, is associated with a lower risk of mortality. Our findings show the value of providing a set of recommendations that reflects a dietary pattern, and contribute to the evidence base to be used when developing sustainable dietary guidelines and policies.

## Supplementary Material

nqab369_Supplemental_File

## Data Availability

Data described in the manuscript can be made available upon request pending application and approval by the chair of the steering committee for the cohort.

## Notes

This study was funded by the Swedish Heart-Lung Foundation (nr 20200482), Crafoord Foundation (nr 20210674), Agenda 2030 Graduate School, Lund University.

The funding agencies had no influence on the design, analysis, or writing of this paper.

Supplemental Tables 1–14 and Supplemental Figures 1–5 are available from the “Supplementary data” link in the online posting of the article and from the same link in the online table of contents at https://academic.oup.com/ajcn/.

Abbreviations used: E%, percentage of the nonalcoholic energy intake; EPIC, European Prospective Investigation into Cancer and Nutrition; ICD-9, International Classification of Diseases, Ninth Revision; ICD-10, International Classification of Diseases, Tenth Revision; MDCS, Malmö Diet and Cancer Study.
