# Supplementary material for: Development of an EAT-Lancet index and its relation to mortality in a Swedish population
Source: Am J Clin Nutr. 2021 Nov 13;115(3):705–16. doi: 10.1093/ajcn/nqab369 (PMC8895215; doi:10.1093/ajcn/nqab369)
Supplement: nqab369_Supplemental_File [file nqab369_supplemental_file.docx]

**Online supplementary material**

This appendix is a part of the original submission and has been peer reviewed.

Supplement to: Stubbendorff A, et al. Development of an EAT-Lancet index and its relation to mortality in a Swedish population.

**Table of contents:**

| Supplemental table 1 | The proposed EAT-Lancet diet by Willett et al., “Food in the Anthropocene: the EAT-Lancet Commission on healthy diets from sustainable food systems”. The diet is based on a daily intake of 2,500 kcal…………………………………………...… | 3 |
| --- | --- | --- |
| Supplemental table 2 | Description of EAT-Lancet index and included food components……………...…. | 4 |
| Supplemental table 3 | Mean intake (grams) and average points (possible range 0-3) by five groups with different adherence to EAT-Lancet diet and by sex in 22,421 participants from the Malmö Diet and Cancer Study……………………………………………………... | 5 |
| Supplemental table 4 | Pearson’s correlations coefficient for intake in grams of food components in 22,421 participants from the Malmö Diet and Cancer Study………………………….…… | 6 |
| Supplemental table 5 | Participant characteristics and EAT-Lancet index in 13,853 women and 8,568 men from the Malmö Diet and Cancer Study……………………………………...…….. | 7 |
| Supplemental table 6 | Associations between EAT-Lancet index and mortality in 22,421 participants from the Malmö Diet and Cancer Study…………………………………………...…….. | 8 |
| Supplemental table 7 | Standardized mortality rates in the EAT-Lancet index categories in 22,421 participants from the Malmö Diet and Cancer Study………………………………. | 9 |
| Supplemental table 8 | Associations between EAT-Lancet index and mortality in 13,853 women from the Malmö Diet and Cancer Study……………………………………………………... | 10 |
| Supplemental table 9 | Associations between EAT-Lancet index and mortality in 8,568 men from the Malmö Diet and Cancer Study………………………………………………….….. | 11 |
| Supplemental table 10 | Associations between EAT-Lancet index and all-cause mortality in 22,421 participants from the Malmö Diet and Cancer Study (post-hoc analyses, 7 categories)………………………………………………………………………… | 12 |
| Supplemental table 11 | Sensitivity analyses of the EAT-Lancet index categories and all-cause mortality in the Malmö Diet and Cancer Study……………………………………………...…... | 13 |
| Supplemental table 12 | Associations between EAT-Lancet index components (14 food groups) and all-cause mortality in 22,388 participants from the Malmö Diet and Cancer Study stratified according to their score………………………………………..…………. | 14 |
| Supplemental table 13 | Associations between EAT-Lancet index components (14 food groups) and cancer mortality in 22,388 participants from the Malmö Diet and Cancer Study stratified according to their score…………………………………………………………….. | 15 |
| Supplemental table 14 | Associations between EAT-Lancet index components (14 food groups) and cardiovascular mortality in 22,388 participants from the Malmö Diet and Cancer Study stratified according to their score……………………………………….…… | 16 |
| Supplemental figure 1 | Flow chart of final sample from the Malmö Diet and Cancer Study……………...… | 17 |
| Supplemental figure 2 | Restricted cubic splines between EAT-Lancet index and included food components (grams per day) and risk of all-cause mortality using Cox regression, based on 22, 421 participants from the Malmö Diet and Cancer Study. Solid line is Hazard Ratio and dotted line is 95% CI………………………………………………..…………. | 18 |
| Supplemental figure 3 | Kaplan-Meier curves showing all-cause mortality for participants stratified by EAT-Lancet index categories, based on 22,421 participants from the Malmö Diet and Cancer Study………………………………………………………….……………. | 19 |
| Supplemental figure 4 | Kaplan-Meier curves showing all-cause mortality for participants stratified by EAT-Lancet index categories, based on 13,853 women from the Malmö Diet and Cancer Study…………………………………………………………………….………… | 19 |
| Supplemental figure 5 | Kaplan-Meier curves showing all-cause mortality for participants stratified by EAT-Lancet index categories, based on 8,568 men from the Malmö Diet and Cancer Study……………………………………………………………………….……… | 19 |
| References for supplemental material...……………………………………………………………………….. | | 20 |

Supplemental table 1. The proposed EAT-Lancet diet by Willett et al., “Food in the Anthropocene: the EAT-Lancet Commission on healthy diets from sustainable food systems” (1). The diet is based on a daily intake of 2,500 kcal.

|  |  | **Macronutrient intake (possible range), g/day** | **Caloric intake, kcal/day** |  |
| --- | --- | --- | --- | --- |
|  | **Whole grains^1^** |  |  |  |
|  | Rice, wheat, corn and other^2^ | 232 (total grains 0-60% of energy) | 811 |  |
|  | **Tubers or starchy vegetables** |  |  |  |
|  | Potatoes and casava | 50 (0-100) | 39 |  |
|  | **Vegetables** |  |  |  |
|  | All vegetables | 300 (200-600) |  |  |
|  | Dark green vegetables | 100 | 23 |  |
|  | Red and orange vegetables | 100 | 30 |  |
|  | Other vegetables | 100 | 25 |  |
|  | **Fruits** |  |  |  |
|  | All fruit | 200 (100-300) | 126 |  |
|  | **Dairy foods** |  |  |  |
|  | Whole milk or derivative equivalents (eg, cheese) | 250 (0-500) | 153 |  |
|  | **Protein sources**^3^ |  |  |  |
|  | Beef and lamb | 7 (0-14) | 15 |  |
|  | Pork | 7 (0-14) | 15 |  |
|  | Chicken and other poultry | 29 (0-58) | 62 |  |
|  | Eggs | 13 (0-25) | 19 |  |
|  | Fish^4^ | 28 (0-100) | 40 |  |
|  | Legumes |  |  |  |
|  | Dry beans, lentils and peas | 50 (0-100) | 172 |  |
|  | Soy foods | 25 (0-50) | 112 |  |
|  | Peanuts | 25 (0-75) | 142 |  |
|  | Tree nuts | 25 | 149 |  |
|  | **Added fats** |  |  |  |
|  | Palm oil | 6.8 (0-6.8) | 60 |  |
|  | Unsaturated oils^5^ | 40 (20-80) | 354 |  |
|  | Dairy fats (included in milk) | 0 | 0 |  |
|  | Lard or tallow^6^ | 5 (0-5) | 36 |  |
|  | **Added sugars** |  |  |  |
|  | All sweeteners | 31 (0-31) | 120 |  |
|  |  |  |  |  |
|  | For an individual, an optimal energy intake to maintain a healthy weight will depend on body size and level of physical activity. Processing of foods such as partial hydrogenation of oils, refining of grains, and addition of salt and preservatives can substantially affect health but is not addressed in this table.  ^1^Wheat, rice, dry beans, and lentils are dry, raw.  ^2^Mix and amount of grains can vary to maintain isocaloric intake.  ^3^Beef and lamb are exchangeable with pork and vice versa. Chicken and other poultry is exchangeable with eggs, fish, or plant protein sources. Legumes, peanuts, tree nuts, seeds, and soy are interchangeable.  ^4^Seafood consist of fish and shellfish (eg, mussels and shrimps) and originate from both capture and from farming. Although seafood is a highly diverse group that contains both animals and plants, the focus of this report is solely on animals.  ^5^Unsaturated oils are 20% each of olive, soybean, rapeseed, sunflower, and peanut oil.  ^6^Some lard or tallow are optional in instances when pigs or cattle are consumed. | | |  |

Supplemental table 2. Description of EAT-Lancet index and included food components.

| **Components in the EAT-Lancet diet index** | |
| --- | --- |
| Whole grains | Fiber-rich breakfast cereals (≥10% fiber), rolled oats, fiber-rich soft bread (>4.5% fiber), fiber-rich crispbread (≥10% fiber), fiber-rich rusks >10% fiber. We evaluate intake of whole grain foods except corn, rice and pasta, because corn was grouped together with vegetables and whole grain alternatives of rice and pasta were grouped together with refined products. In order to adjust for not including corn, rice and pasta and still relate the intake levels in MDC to the suggested EAT-Lancet reference values, intake of whole grain was divided by 0.75 based on the observation that corn, rice and pasta contribute to 25% of total cereal intake in an ongoing study in Malmö (2). |
| Potatoes | Boiled potatoes, fried potatoes, deep fried potatoes, potatoes included in dishes such as potato salad and moussaka. |
| Vegetables | All vegetables except legumes. |
| Fruits | Fruits and berries. |
| Dairy | Whole milk or derivative equivalents. Regular milk, low-fat milk, yoghurt and other fermented milk products, hard cheese, soft cheese, cream, butter, butter-based spreads. In the EAT-Lancet diet all dairy foods are expressed as of milk equivalents. The milk equivalents we used are based on the approach used by Stockholm Resilience Centre, based on ‘total solids’ and intakes of different dairy products were consequently multiplied with the following factors; whole milk 1.0, Cheese 5.0, cream 2.7 and butter 6.5 (3). |
| Beef and lamb | Beef, lamb, minced meat with pork and lamb, processed meats with beef and lamb including sausages. |
| Pork | Pork, minced meat of pork, processed meats with pork including ham, bacon, and sausages. |
| Chicken | Chicken, turkey, duck, goose, and other poultry. |
| Eggs | Boiled eggs, fried eggs and eggs in dishes such as omelet and pie. |
| Fish | Fatty fish, lean fish, fish products, shellfish. |
| Legumes | Dry beans, lentils, peas, soy. Targets and index refer to raw weight. Peas, lentils, beans, tofu, soy containing meat replacement products. |
| Nuts | Peanuts or tree nuts. All nuts and seeds including peanuts, nut mixes such as almond paste. |
| Unsaturated oils | All plant oils and plant margarines. |
| Added sugar | Sucrose and monosaccharides except sugars in fruits and vegetables (4). |

**Supplemental table 3**. Mean intake (grams) and average points (possible range 0-3) by five groups with different adherence to EAT-Lancet diet and by sex in 22,421 participants from the Malmö Diet and Cancer Study^1^.

|  | **Mean intake (g) across EAT-Lancet index categories** | | | | | | | |  | |
| --- | --- | --- | --- | --- | --- | --- | --- | --- | --- | --- |
|  | ≤13 | 14-16 | 17-19 | 20-22 | ≥23 | All | Men | Women | | Target (reference interval) in EAT-Lancet diet (g) |
| Whole grains | 45.35 (±46.96) | 59.38 (±57.95) | 72.04 (±58.64) | 89.64 (±70.82) | 111.95 (±83.84) | 73.60 (±65.19) | 85.10 (±79.42) | 66.48 (±53.35) | | 232 |
| Potatoes | 164.84 (±88.45) | 140.74 (±75.45) | 121.82 (±68.38) | 99.75 (±61.13) | 79.73 (±59.32) | 122.18 (±73.78) | 155.99 (±85.74) | 101.27 (±55.84) | | 50 (0-100) |
| Vegetables | 106.83 (±59.38) | 133.86 (±73.8) | 161.04 (±83.5) | 191.37 (±98.21) | 243.37 (±128.16) | 162.73 (±94.13) | 153.76 (±92.75) | 168.28 (±94.55) | | 300 (200-600) |
| Fruits | 98.87 (±87.54) | 150.56 (±108.42) | 196.08 (±117.57) | 234.8 (±117.52) | 283.68 (±152.53) | 191.47 (±125.91) | 169.96 (±128.51) | 204.78 (±122.40) | | 200 (100-300) |
| Dairy | 930.01 (±408.95) | 780.68 (±367.46) | 679.33 (±311.16) | 613.13 (±295.37) | 587.22 (±337.79) | 705.83 (±348.89) | 757.92 (±395.06) | 673.61 (±312.69) | | 250 (0-500) |
| Beef and lamb | 60.05 (±32.70) | 51.35 (±30.88) | 44.47 (±28.56) | 36.33 (±27.19) | 23.17 (±25.31) | 44.08 (±30.48) | 56.08 (±35.35) | 36.66 (±24.23) | | 7 (0-14) |
| Pork | 84.67 (±47.67) | 70.9 (±39.89) | 62.74 (±36.18) | 52.04 (±34.14) | 34.73 (±33.28) | 62.16 (±39.68) | 78.46 (±45.89) | 52.08 (±31.30) | | 7 (0-14) |
| Poultry | 20.39 (±27.86) | 18.12 (±24.27) | 15.59 (±20.81) | 14.68 (±19.58) | 12.97 (±18.17) | 16.25 (±22.10) | 17.54 (±24.78) | 15.45 (±20.23) | | 29 (0-58) |
| Eggs | 33.01 (±23.45) | 27.42 (±20.05) | 22.87 (±17.58) | 18.44 (±15.61) | 14.98 (±13.79) | 23.32 (±18.84) | 25.81 (±21.01) | 21.78 (±17.18) | | 13 (0-25) |
| Fish | 29.13 (±34.2) | 39.49 (±34) | 46.82 (±33.72) | 51.39 (±32.67) | 55.36 (±38.24) | 45.05 (±34.74) | 49.57 (±40.30) | 42.25 (±30.47) | | 28 (0-100) |
| Legumes | 5.43 (±8.48) | 5.66 (±8.41) | 5.98 (±8.68) | 6.60 (±9.40) | 9.63 (±22.16) | 6.29 (±10.57) | 7.83 (±11.11) | 5.33 (±10.11) | | 75 (0-150) |
| Nuts | 1.48 (±3.48) | 1.62 (±3.81) | 1.71 (±4.42) | 2.02 (±5.47) | 3.25 (±8.77) | 1.86 (±4.98) | 2.23 (±5.65) | 1.63 (±4.50) | | 50 (0-100) |
| Unsaturated oils | 20.75 (±23.35) | 27.68 (±24.56) | 31.74 (±23.60) | 33.13 (±22.44) | 33.51 (±22.8) | 30.15 (±23.78) | 37.19 (±28.58) | 25.79 (±18.99) | | 40 (20-80) |
| Added sugar | 76.32 (±39.9) | 64.6 (±34.27) | 54.97 (±29.02) | 47.79 (±26.99) | 41.59 (±24.61) | 56.67 (±32.26) | 66.32 (±35.83) | 50.7 (±28.24) | | 31 (0-31) |
|  |  |  |  |  |  |  |  |  | |  |
|  | **Average points in each group across EAT-Lancet index categories** | | | | | | | | |  |
| Whole grains | 0.33 (±0.61) | 0.50 (±0.73) | 0.70 (±0.79) | 0.95 (±0.86) | 1.24 (±0.89) | 0.72 (±0.82) | 0.85 (±0.90) | 0.63 (±0.75) | |  |
| Potatoes | 0.95 (±0.76) | 1.19 (±0.78) | 1.41 (±0.80) | 1.72 (±0.81) | 2.04 (±0.82) | 1.43 (±0.85) | 1.07 (±0.80) | 1.65 (±0.80) | |  |
| Vegetables | 0.53 (±0.63) | 0.80 (±0.74) | 1.09 (±0.81) | 1.39 (±0.88) | 1.80 (±0.91) | 1.09 (±0.87) | 1.00 (±0.87) | 1.15 (±0.87) | |  |
| Fruits | 1.12 (±0.97) | 1.74 (±0.96) | 2.17 (±0.84) | 2.49 (±0.68) | 2.72 (±0.52) | 2.08 (±0.94) | 1.86 (±1.00) | 2.21 (±0.88) | |  |
| Dairy | 0.72 (±0.68) | 1.00 (±0.71) | 1.21 (±0.73) | 1.39 (±0.76) | 1.52 (±0.83) | 1.18 (±0.77) | 1.10 (±0.80) | 1.23 (±0.74) | |  |
| Beef and lamb | 0.12 (±0.37) | 0.27 (±0.58) | 0.40 (±0.69) | 0.72 (±0.91) | 1.48 (±1.19) | 0.50 (±0.83) | 0.29 (±0.66) | 0.63 (±0.89) | |  |
| Pork | 0.05 (±0.24) | 0.10 (±0.36) | 0.16 (±0.46) | 0.39 (±0.75) | 1.10 (±1.21) | 0.26 (±0.66) | 0.14 (±0.50) | 0.34 (±0.73) | |  |
| Poultry | 2.57 (±0.69) | 2.65 (±0.62) | 2.74 (±0.53) | 2.78 (±0.49) | 2.83 (±0.44) | 2.72 (±0.56) | 2.67 (±0.61) | 2.75 (±0.52) | |  |
| Eggs | 1.39 (±0.99) | 1.64 (±0.97) | 1.90 (±0.94) | 2.18 (±0.88) | 2.40 (±0.81) | 1.89 (±0.97) | 1.78 (±1.01) | 1.96 (±0.94) | |  |
| Fish | 1.59 (±1.29) | 2.14 (±1.12) | 2.49 (±0.89) | 2.69 (±0.70) | 2.70 (±0.73) | 2.38 (±1.00) | 2.40 (±1.01) | 2.37 (±1.00) | |  |
| Legumes | 0.08 (±0.30) | 0.08 (±0.32) | 0.10 (±0.33) | 0.12 (±0.38) | 0.21 (±0.53) | 0.11 (±0.36) | 0.17 (±0.44) | 0.07 (±0.29) | |  |
| Nuts | 0.02 (±0.14) | 0.02 (±0.18) | 0.03 (±0.22) | 0.05 (±0.28) | 0.12 (±0.42) | 0.04 (±0.24) | 0.05 (±0.28) | 0.03 (±0.22) | |  |
| Unsaturated oils | 0.98 (±1.09) | 1.45 (±1.14) | 1.75 (±1.07) | 1.88 (±1.01) | 1.92 (±1.01) | 1.64 (±1.11) | 1.87 (±1.15) | 1.51 (±1.06) | |  |
| Added sugar | 1.34 (±0.78) | 1.60 (±0.78) | 1.84 (±0.75) | 2.05 (±0.74) | 2.25 (±0.72) | 1.81 (±0.80) | 1.59 (±0.80) | 1.95 (±0.76) | |  |
| ^1^Values are means and SD. | | | | | | | | | | |

**Supplemental table 4**. Pearson’s correlations coefficient for intake in grams of food components in 22,421 participants from the Malmö Diet and Cancer Study.

|  | Added sugar | Beef and lamb | EAT-Lancet Score | Egg | Fish | Fruit | Legumes | Dairy | Nuts | Unsatura-ted oils | Pork | Potatoes | Poultry | Vegetables | |
| --- | --- | --- | --- | --- | --- | --- | --- | --- | --- | --- | --- | --- | --- | --- | --- |
| Added sugar |  |  |  |  |  |  |  |  |  |  |  |  |  |  |  |
| Beef and lamb | 0.129^**^ |  |  |  |  |  |  |  |  |  |  |  |  |  |  |
| EAT-Lancet Score | -0.301^1^ | -0.320^1^ |  |  |  |  |  |  |  |  |  |  |  |  |  |
| Egg | 0.078^1^ | 0.108^1^ | -0.273^1^ |  |  |  |  |  |  |  |  |  |  |  |  |
| Fish | 0.020^1^ | -0.022^1^ | 0.208^1^ | 0.150^1^ |  |  |  |  |  |  |  |  |  |  |  |
| Fruit | -00.003 | -0.086^1^ | 0.401^1^ | 0.000 | 0.089^1^ |  |  |  |  |  |  |  |  |  |  |
| Legumes | 0.053^1^ | 0.026^1^ | 0.104^1^ | 0.017^2^ | 0.028^1^ | 0.051^1^ |  |  |  |  |  |  |  |  |  |
| Dairy | 0.252^1^ | 0.065^1^ | -0.284^1^ | 0.111^1^ | 0.057^1^ | 0.019^1^ | 0.027^1^ |  |  |  |  |  |  |  |  |
| Nuts | 0.058^1^ | 0.022^1^ | 0.090^1^ | 0.014^2^ | 0.018^1^ | 0.079^1^ | 0.049^1^ | 00.011 |  |  |  |  |  |  |  |
| Unsaturated oils | 0.187^1^ | 0.136^1^ | 0.144^1^ | 0.085^1^ | 0.049^1^ | -0.051^1^ | 0.040^1^ | -0.033^1^ | -00.004 |  |  |  |  |  |  |
| Pork | 0.167^1^ | 0.280^1^ | -0.331^1^ | 0.155^1^ | -0.064^1^ | -0.128^1^ | 0.108^1^ | 0.070^1^ | -0.021^1^ | 0.180^1^ |  |  |  |  |  |
| Potatoes | 0.186^1^ | 0.318^1^ | -0.322^1^ | 0.123^1^ | 0.184^1^ | -0.082^1^ | 0.055^1^ | 0.108^1^ | 0.008 | 0.199^1^ | 0.392^1^ |  |  |  |  |
| Poultry | -0.026^1^ | -0.024^1^ | -0.096^1^ | 0.013 | 0.026^1^ | 0.034^1^ | -0.023^1^ | -0.018^1^ | 0.022^1^ | -0.015^2^ | -0.055^1^ | 0.033^1^ |  |  |  |
| Vegetables | -0.069^1^ | 0.045^1^ | 0.387^1^ | 0.081^1^ | 0.153^1^ | 0.318^1^ | 0.083^1^ | 0.022^1^ | 0.094^1^ | -0.020^1^ | -0.028^1^ | -00.003 | 0.089^1^ |  |  |
| Whole grains | 0.111^1^ | 0.010 | 0.279^1^ | 0.024^1^ | 0.111^1^ | 0.126^1^ | 0.054^1^ | 0.151^1^ | 0.013^2^ | 0.153^1^ | -00.010 | 0.077^1^ | 00.009 | 0.141^1^ |  |
| ^1^p-value <0.001  ^2^p-vaule <0.05 | | | | | | | | | | | | | | | |

Supplemental table 5. Participant characteristics and EAT-Lancet index in 13,853 women and 8,568 men from the Malmö Diet and Cancer Study1.

|  | **EAT-Lancet index categories** | | | | |  |
| --- | --- | --- | --- | --- | --- | --- |
| **Women** | **≤13** | **14-16** | **17-19** | **20-22** | **≥23** | **P-trend^2^** |
| Age (years) | 55.06 (54.53, 55.59) | 56.28 (55.99, 56.57) | 57.21 (56.99, 57.42) | 57.63 (57.37, 57.89) | 57.23 (56.82, 57.64) | <0.001 |
| BMI (kg/m^2^)^3^ | 24.85 (24.58, 25.13) | 25.26 (25.11, 25.41) | 25.48 (25.37, 25.59) | 25.36 (25.23, 25.49) | 24.73 (24.52, 24.94) | 0.28 |
| Energy intake (kcal)^4^ | 2,265 (2,233, 2,298) | 2,151 (2,133, 2,169) | 2,046 (2,033, 2,060) | 1,946 (1,931, 1,962) | 1,901 (1,876, 1,926) | <0.001 |
| Fat (E%)^4^ | 41.71 (41.32, 42.09) | 39.68 (39.47, 39.90) | 38.10 (37.94, 38.25) | 36.35 (36.16, 36.54) | 35.00 (34.70, 35.30) | <0.001 |
| Carbohydrates (E%)^4^ | 43.57 (43.18, 43.96) | 45.02 (44.81, 45.24) | 46.22 (46.06, 46.38) | 47.76 (47.57, 47.95) | 49.51 (49.21, 49.81) | <0.001 |
| Protein (E%)^4^ | 14.72 (14.56, 14.89) | 15.29 (15.20, 15.38) | 15.69 (15.62, 15.76) | 15.89 (15.81, 15.97) | 15.49 (15.36, 15.62) | <0.001 |
| Dietary fibre (g)^4^ | 14.68 (14.29, 15.06) | 16.63 (16.42, 16.84) | 18.46 (18.3, 18.62) | 20.28 (20.09, 20.46) | 23.41 (23.11, 23.70) | <0.001 |
| Dietary fibre (g)/MJ^4^ | 1.57 (1.53, 1.60) | 1.86 (1.84, 1.88) | 2.17 (2.16, 2.19) | 2.51 (2.49, 2.53) | 2.98 (2.95, 3.00) | <0.001 |
|  |  |  |  |  |  |  |
|  |  |  |  |  |  | P-value^5^ |
| Overweight (BMI 25-29.99) | 30.9% | 31.6% | 34.4% | 34.2% | 30.2% | <0.001 |
| Obese (BMI >30) | 10.3% | 12.8% | 13.7% | 12.4% | 10.1% | <0.001 |
| BMI <25 | 58.8% | 55.6% | 51.9% | 53.4% | 59.7% | <0.001 |
| Current smoker | 50.1% | 36.5% | 26.3% | 22.6% | 21.3% | <0.001 |
| High alcohol intake^6^ | 3.3% | 3.0% | 2.3% | 1.9% | 2.7% | <0.001 |
| University degree | 10.3% | 13.8% | 15.1% | 17.3% | 20.6% | <0.001 |
| High physical activity^7^ | 14.1% | 16.9% | 20.0% | 22.7% | 26.5% | <0.001 |
|  |  |  |  |  |  |  |
| **Men** |  |  |  |  |  | p-trend^2^ |
| Age (years) | 57.62 (57.25, 57.99) | 58.71 (58.43, 58.98) | 59.12 (58.96, 59.47) | 59.34 (58.97, 59.70) | 58.53 (57.82, 59.24) | <0.001 |
| BMI (kg/m^2^)^3^ | 25.82 (25.64, 26.00) | 26.05 (25.91, 26.18) | 26.26 (26.13, 26.38) | 26.20 (26.02, 26.37) | 26.27 (25.92, 26.61) | <0.001 |
| Energy intake (kcal)^4^ | 2,912 (2,877, 2,947) | 2,732 (2,707, 2,758) | 2,598 (2,574, 2,621) | 2,514 (2,480, 2,549) | 2,421 (2,355, 2,487) | <0.001 |
| Fat (E%)^4^ | 42.37 (42.05, 42.69) | 40.22 (39.99, 40.45) | 38.70 (38.48, 38.92) | 36.95 (36.64, 37.26) | 34.83 (34.22, 35.43) | <0.001 |
| Carbohydrates (E%)^4^ | 43.02 (42.70, 43.35) | 44.70 (44.46, 44.93) | 45.98 (45.76, 46.19) | 47.66 (47.35, 47.98) | 49.82 (49.20, 50.43) | <0.001 |
| Protein (E%)^4^ | 14.61 (14.48, 14.74) | 15.08 (14.99, 15.18) | 15.32 (15.24, 15.41) | 15.39 (15.26, 15.51) | 15.36 (15.12, 15.60) | <0.001 |
| Dietary fibre (g)^4^ | 17.84 (17.46, 18.22) | 19.85 (19.58, 20.12) | 21.80 (21.55, 22.06) | 24.56 (24.19, 24.92) | 29.72 (29.00, 30.43) | <0.001 |
| Dietary fibre (g)/MJ^4^ | 1.48 (1.45, 1.50) | 1.74 (1.72, 1.76) | 2.01 (2.00, 2.03) | 2.35 (2.32, 2.37) | 2.94 (2.90, 2.99) | <0.001 |
|  |  |  |  |  |  |  |
|  |  |  |  |  |  | P-value^5^ |
| Overweight (BMI 25-29.99) | 46.8% | 48.6% | 50.1% | 51.3% | 49.6% | <0.001 |
| Obese (BMI >30) | 10.9% | 11.4% | 12.6% | 11.1% | 12.5% | <0.001 |
| BMI <25 | 42.3% | 40% | 37.3% | 37.6% | 37.9% | <0.001 |
| Current smoker | 45.6% | 34.3% | 25.0% | 18.0% | 17.3% | <0.001 |
| High alcohol intake^6^ | 9.6% | 9.0% | 7.1% | 5.8% | 6.1% | <0.001 |
| University degree | 9.9% | 12.7% | 14.3% | 16.3% | 19.2% | <0.001 |
| High physical activity^7^ | 15.1% | 19.5% | 19.8% | 22.9% | 25.9% | <0.001 |
|  |  |  |  |  |  |  |
| ^1^Values are means or percentages. ^2^General linear model, P-trend across index categories. ^3^Adjusted for age. ^4^Adjusted for age, dietary assessment version, and season. ^5^Chi-2 test. ^6^High alcohol consumption defined as above 30 g/d for women and above 40g/d for men. ^7^Highest leisure time physical activity quintile. | | | | | | |

Supplemental table 6. Associations between EAT-Lancet index and mortality in 22,421 participants from the Malmö Diet and Cancer Study1.

|  | | **EAT-Lancet index categories** | | | | | | | | | | | | | | | |  |
| --- | --- | --- | --- | --- | --- | --- | --- | --- | --- | --- | --- | --- | --- | --- | --- | --- | --- | --- |
| **All-cause mortality** | | | **≤13** | | **14-16** | | | **17-19** | | | **20-22** | | | **≥23** | | | **P-trend2** | |
| n/cases | 2,197/807 | | | 5,381/1,833 | | | 7,977/2,484 | | | 5,037/1,444 | | | 1,829/462 | | |  | | |
| Person years (sum) | 42,474 | | | 105,972 | | | 160,017 | | | 102,834 | | | 38,123 | | |  | | |
| Model 13 | 1 | | | 0.82 (0.75, 0.89) | | | 0.71 (0.65, 0.77) | | | 0.64 (0.59, 0.70) | | | 0.59 (0.53, 0.67) | | | 1.2E-28 | | |
| Model 2^4^ | 1 | | | 0.90 (0.83, 0.98) | | | 0.84 (0.77, 0.91) | | | 0.79 (0.72, 0.87) | | | 0.75 (0.66, 0.84) | | | 6.2E-09 | | |
| Model 35 | 1 | | | 0.90 (0.83, 0.98) | | | 0.83 (0.77, 0.91) | | | 0.79 (0.72, 0.87) | | | 0.75 (0.67, 0.85) | | | 5.7E-09 | | |
| Model 46 | 1 | | | 0.91 (0.83, 0.98) | | | 0.85 (0.78, 0.92) | | | 0.8 (0.73, 0.88) | | | 0.76 (0.67, 0.85) | | | 1.3E-08 | | |
|  |  | | |  | | |  | | |  | | |  | | |  | | |
| **Cancer mortality7** | | | |  | | |  | | |  | | |  | | |  | | |
| n/cases | 2,191/317 | | | 5,372/689 | | | 7,970/936 | | | 5,029/535 | | | 1,829/178 | | |  | | |
| Person years (sum) | 42,369 | | | 105,822 | | | 159,901 | | | 102725 | | | 38,069 | | |  | | |
| Model 13 | 1 | | | 0.82 (0.72, 0.94) | | | 0.72 (0.63, 0.82) | | | 0.64 (0.56, 0.74) | | | 0.60 (0.50, 0.73) | | | 8.3E-15 | | |
| Model 2^4^ | 1 | | | 0.91 (0.79, 1.04) | | | 0.86 (0.75, 0.98) | | | 0.80 (0.69, 0.92) | | | 0.76 (0.63, 0.92) | | | 9.0E-06 | | |
| Model 35 | 1 | | | 0.90 (0.79, 1.03) | | | 0.85 (0.75, 0.97) | | | 0.79 (0.68, 0.92) | | | 0.76 (0.63, 0.92) | | | 1.0E-05 | | |
| Model 46 | 1 | | | 0.91 (0.80, 1.04) | | | 0.87 (0.76, 0.99) | | | 0.81 (0.70, 0.94) | | | 0.78 (0.64, 0.94) | | | 1.3E-07 | | |
|  |  | | |  | | |  | | |  | | |  | | |  | | |
| **Cardiovascular mortality^7^** | | | | | |  | | |  | | |  | | |  | | | |
| n/cases | 2,191/266 | | | 5,372/577 | | | 7,970/776 | | | 5,029/441 | | | 1,829/132 | | |  | | |
| Person years (sum) | 42,369 | | | 105,822 | | | 159,901 | | | 102,725 | | | 38,069 | | |  | | |
| Model 13 | 1 | | | 0.76 (0.66, 0.88) | | | 0.66 (0.57, 0.76) | | | 0.59 (0.50, 0.69) | | | 0.53 (0.43, 0.66) | | | 8.6E-12 | | |
| Model 2^4^ | 1 | | | 0.84 (0.73, 0.97) | | | 0.78 (0.67, 0.90) | | | 0.73 (0.62, 0.86) | | | 0.68 (0.54, 0.84) | | | 4.9E-09 | | |
| Model 35 | 1 | | | 0.83 (0.72, 0.97) | | | 0.76 (0.66, 0.88) | | | 0.72 (0.62, 0.85) | | | 0.68 (0.54, 0.84) | | | 7.7E-09 | | |
| Model 46 | 1 | | | 0.85 (0.73, 0.98) | | | 0.79 (0.68, 0.91) | | | 0.74 (0.63, 0.87) | | | 0.69 (0.55, 0.85) | | | 6.9E-14 | | |
| ^1^Values are HR, 95% CI. Multivariate proportional hazards were used to examine the associations. ^2^P-trend across index categories. ^3^Adjusted for age, sex, dietary assessment version , season, energy intake. ^4^As above plus physical activity, smoking, alcohol use and education. ^5^As above plus BMI. ^6^Adjusted for age, sex, dietary assessment version, season, physical activity, smoking, alcohol use and education. ^7^33 participants with missing values about death cause were excluded (22,388 included). | | | | | | | | | | | | | | | | | | |

Supplemental table 7. Standardized mortality rates in the EAT-Lancet index categories in 22,421 participants from the Malmö Diet and Cancer Study1.

|  | | **EAT-Lancet index categories** | | | | |  | |
| --- | --- | --- | --- | --- | --- | --- | --- | --- |
| **All-cause mortality** | **≤13** | | 14-16 | 17-19 | **20-22** | **≥23** | |  |
| n/cases | 2,197/807 | | 5,381/1,833 | 7,977/2,484 | 5,037/1,444 | 1,829/462 | |  |
| 10-y mortality risk (%) | 5.1 (4.7, 5.5) | | 4.5 (4.2, 4.9) | 4.1 (3.9, 4.4) | 3.9 (3.6, 4.2) | 3.7 (3.3, 4.1) | |  |
| 15-y mortality risk (%) | 11.4 (10.6, 12.3) | | 10.2 (9.7, 10.8) | 9.3 (8.6, 9.8) | 8.8 (8.3, 9.3) | 8.4 (7.6, 9.2) | |  |
| 20-y mortality risk (%) | 21.9 (20.5, 23.4) | | 19.8 (18.8, 20.7) | 18.1 (17.4, 18.9) | 17.1 (16.2, 18.0) | 16.4 (15.0, 17.8) | |  |
| ^1^Values are standardized mortality rates, 95% CI. Cox regression model adjusted for age, sex, dietary assessment version, season, energy intake, physical activity, smoking, alcohol use, education, and BMI. | | | | | | | |  |

Supplemental table 8. Associations between EAT-Lancet index and mortality in 13,853 women from the Malmö Diet and Cancer Study1.

|  | | | **EAT-Lancet index categories** | | | | | | | | | | | | | | | | | | |  |  | |  |
| --- | --- | --- | --- | --- | --- | --- | --- | --- | --- | --- | --- | --- | --- | --- | --- | --- | --- | --- | --- | --- | --- | --- | --- | --- | --- |
| **All-cause mortality** | | | | **≤13** | | **14-16** | | | **17-19** | | | **20-22** | | | **≥23** | | |  | | | **P-trend2** | | | |  |
| n/cases | | 857/241 | | | 2,851/789 | | | 5,044/1,286 | | | 3,647/912 | | | 1,454/342 | | |  | | |  | | | | |  |
| Person years (sum) | | 17,138 | | | 57,450 | | | 103,226 | | | 75,443 | | | 30,521 | | |  | | |  | | | | |  |
| Model 13 | | 1 | | | 0.80 (0.69, 0.92) | | | 0.64 (0.55, 0.73) | | | 0.59 (0.51, 0.68) | | | 0.56 (0.47, 0.66) | | |  | | | 1.9E-17 | | | | |  |
| Model 2^4^ | | 1 | | | 0.87 (0.76, 1.01) | | | 0.77 (0.67, 0.88) | | | 0.73 (0.63, 0.84) | | | 0.70 (0.59, 0.83) | | |  | | | 4.7E-07 | | | | |  |
| Model 35 | | 1 | | | 0.87 (0.75, 1.00) | | | 0.76 (0.66, 0.87) | | | 0.73 (0.63, 0.84) | | | 0.70 (0.59, 0.84) | | |  | | | 7.1E-07 | | | | |  |
| Model 46 | | 1 | | | 0.88 (0.76, 1.01) | | | 0.77 (0.67, 0.88) | | | 0.73 (0.63, 0.85) | | | 0.71 (0.6, 0.84) | | |  | | | 3.5E-07 | | | | |  |
|  | |  | | |  | | |  | | |  | | |  | | |  | | |  | | | | |  |
| **Cancer mortality7** | | | | |  | | |  | | |  | | |  | | |  | | |  | | | | |  |
| n/cases | | 856/99 | | | 2,849/320 | | | 5,043/497 | | | 3,644/341 | | | 1,451/139 | | |  | | |  | | | | |  |
| Person years (sum) | | 17,122 | | | 57,408 | | | 103,206 | | | 75,405 | | | 30,467 | | |  | | |  | | | | |  |
| Model 13 | | 1 | | | 0.86 (0.69, 1.08) | | | 0.69 (0.55, 0.86) | | | 0.62 (0.50, 0.78) | | | 0.64 (0.49, 0.83) | | |  | | | 1.0E-06 | | | | |  |
| Model 2^4^ | | 1 | | | 0.96 (0.76, 1.2) | | | 0.83 (0.67, 1.03) | | | 0.78 (0.62, 0.98) | | | 0.8 (0.62, 1.05) | | |  | | | 7.7E-03 | | | | |  |
| Model 35 | | 1 | | | 0.95 (0.76, 1.19) | | | 0.82 (0.66, 1.02) | | | 0.77 (0.61, 0.97) | | | 0.81 (0.62, 1.05) | | |  | | | 8.5E-03 | | | | |  |
| Model 46 | | 1 | | | 0.96 (0.76, 1.2) | | | 0.83 (0.67, 1.04) | | | 0.78 (0.62, 0.98) | | | 0.81 (0.62, 1.05) | | |  | | | 6.9E-03 | | | | |  |
|  | |  | | |  | | |  | | |  | | |  | | |  | | |  | | | | |  |
| **Cardiovascular mortality^7^** | | | | | | |  | | |  | | |  | | |  | | |  | | | | | |  |
| n/cases | | 856/65 | | | 2849/218 | | | 5043/366 | | | 3644/264 | | | 1451/90 | | |  | | |  | | | | |  |
| Person years (sum) | | 17,122 | | | 57,408 | | | 103,206 | | | 75,405 | | | 30,467 | | |  | | |  | | | | |  |
| Model 13 | | 1 | | | 0.75 (0.57, 0.99) | | | 0.6 (0.46, 0.78) | | | 0.56 (0.42, 0.73) | | | 0.49 (0.35, 0.68) | | |  | | | 6.3E-07 | | | | |  |
| Model 2^4^ | | 1 | | | 0.81 (0.62, 1.08) | | | 0.71 (0.54, 0.93) | | | 0.68 (0.51, 0.90) | | | 0.61 (0.44, 0.85) | | |  | | | 1.3E-03 | | | | |  |
| Model 35 | | 1 | | | 0.81 (0.61, 1.07) | | | 0.70 (0.53, 0.92) | | | 0.68 (0.51, 0.89) | | | 0.62 (0.45, 0.87) | | |  | | | 2.1E-03 | | | | |  |
| Model 46 | | 1 | | | 0.81 (0.62, 1.08) | | | 0.71 (0.54, 0.93) | | | 0.68 (0.51, 0.89) | | | 0.61 (0.44, 0.85) | | |  | | | 9.2E-04 | | | | |  |
|  | ^1^Values are HR, 95% CI. Multivariate proportional hazards were used to examine the associations. ^2^P-trend across index categories. ^3^Adjusted for age, sex, dietary assessment version, season, energy intake. ^4^As above plus physical activity, smoking, alcohol use and education. ^5^As above plus BMI. ^6^Adjusted for age, sex, dietary assessment version, season, physical activity, smoking, alcohol use and education. ^7^10 participants with missing values about death cause were excluded (13,843 included). | | | | | | | | | | | | | | | | | | | | | | |  |  |

Supplemental table 9. Associations between EAT-Lancet index and mortality in 8,568 men from the Malmö Diet and Cancer Study1.

|  | | **EAT-Lancet index categories** | | | | | | | | | |  |  |
| --- | --- | --- | --- | --- | --- | --- | --- | --- | --- | --- | --- | --- | --- |
| **All-cause mortality** | **≤13** | | **14-16** | | **17-19** | | **20-22** | | **≥23** | | **P-trend2** | |  |
| n/cases | 1,340/566 | | 2,530/1,044 | | 2,933/1,198 | | 1,390/532 | | 375/120 | |  | |  |
| Person years (sum) | 25,336 | | 48,522 | | 56,791 | | 27,391 | | 7,602 | |  | |  |
| Model 13 | 1 | | 0.82 (0.74, 0.91) | | 0.76 (0.68, 0.84) | | 0.68 (0.6, 0.76) | | 0.59 (0.48, 0.72) | | 4.9E-13 | |  |
| Model 2^4^ | 1 | | 0.91 (0.82, 1.00) | | 0.89 (0.8, 0.98) | | 0.85 (0.75, 0.96) | | 0.75 (0.61, 0.92) | | 1.5E-03 | |  |
| Model 35 | 1 | | 0.90 (0.82, 1.00) | | 0.88 (0.8, 0.98) | | 0.84 (0.74, 0.95) | | 0.74 (0.61, 0.91) | | 1.1E-03 | |  |
| Model 46 | 1 | | 0.91 (0.82, 1.01) | | 0.9 (0.81, 1) | | 0.86 (0.76, 0.97) | | 0.76 (0.62, 0.93) | | 3.4E-03 | |  |
|  |  | |  | |  | |  | |  | |  | |  |
| **Cancer mortality7** | | |  | |  | |  | |  | |  | |  |
| n/cases | 1,335/218 | | 2,523/369 | | 2,927/439 | | 1,385/194 | | 375/39 | |  | |  |
| Person years (sum) | 25,247 | | 48,414 | | 56,695 | | 27,320 | | 7,602 | |  | |  |
| Model 13 | 1 | | 0.77 (0.65, 0.92) | | 0.75 (0.63, 0.88) | | 0.66 (0.54, 0.81) | | 0.50 (0.36, 0.71) | | 3.0E-06 | |  |
| Model 2^4^ | 1 | | 0.86 (0.72, 1.02) | | 0.88 (0.74, 1.04) | | 0.83 (0.68, 1.02) | | 0.64 (0.45, 0.90) | | 3.0E-02 | |  |
| Model 35 | 1 | | 0.86 (0.72, 1.02) | | 0.88 (0.74, 1.04) | | 0.83 (0.68, 1.02) | | 0.64 (0.45, 0.90) | | 2.9E-02 | |  |
| Model 46 | 1 | | 0.87 (0.73, 1.02) | | 0.9 (0.76, 1.06) | | 0.86 (0.70, 1.04) | | 0.66 (0.47, 0.93) | | 5.5E-02 | |  |
|  |  | |  | |  | |  | |  | |  | |  |
| **Cardiovascular mortality^7^** | | | |  | |  | |  | |  | | |  |
| n/cases | 1,335/201 | | 2,523/359 | | 2,927/410 | | 1,385/177 | | 375/42 | |  | |  |
| Person years (sum) | 25,247 | | 48,414 | | 56,695 | | 27,320 | | 7,602 | |  | |  |
| Model 13 | 1 | | 0.77 (0.65, 0.92) | | 0.70 (0.59, 0.83) | | 0.60 (0.49, 0.74) | | 0.56 (0.40, 0.78) | | 2.9E-07 | |  |
| Model 2^4^ | 1 | | 0.85 (0.72, 1.02) | | 0.82 (0.69, 0.98) | | 0.76 (0.62, 0.94) | | 0.72 (0.51, 1.01) | | 7.8E-03 | |  |
| Model 35 | 1 | | 0.85 (0.71, 1.01) | | 0.81 (0.68, 0.96) | | 0.75 (0.61, 0.93) | | 0.70 (0.50, 0.99) | | 4.1E-03 | |  |
| Model 46 | 1 | | 0.86 (0.73, 1.03) | | 0.84 (0.71, 1.00) | | 0.79 (0.64, 0.97) | | 0.75 (0.54, 1.05) | | 1.8E-02 | |  |
| ^1^Values are HR, 95% CI. Multivariate proportional hazards were used to examine the associations. ^2^P-trend across index categories. ^3^adjusted for age, sex, dietary assessment version, season, energy intake. ^4^As above plus physical activity, smoking, alcohol use and education. ^5^As above plus BMI. ^6^Adjusted for age, sex, dietary assessment version, season, physical activity, smoking, alcohol use and education. ^7^23 participants with missing values about death cause were excluded (8,545 included). | | | | | | | | | | | | |  |

Supplemental table 10. Associations between EAT-Lancet index and all-cause mortality in 22,421 participants from the Malmö Diet and Cancer Study (post-hoc analyses, 7 categories)^1^.

|  |  | | **EAT-Lancet index categories** | | | | | |  | |  | |
| --- | --- | --- | --- | --- | --- | --- | --- | --- | --- | --- | --- | --- |
| **All-cause mortality** | | **≤10** | | **11-13** | **14-16** | **17-19** | **20-22** | **23-25** | | **≥26** | | **P-trend^2^** |
| n/cases | | 369/157 | | 1,828/650 | 5,981/1,833 | 7,977/2,484 | 3,864/1,107 | 2,406/669 | | 596/130 | |  |
| Person years (sum) | | 7,082 | | 35,392 | 105,972 | 160,017 | 78,820 | 49,465 | | 12,672 | |  |
| Model 1^3^ | | 1 | | 0.78 (0.65, 0.93) | 0.67 (0.57, 0.78) | 0.57 (0.49, 0.68) | 0.52 (0.44, 0.62) | 0.51 (0.43, 0.61) | | 0.44 (0.34, 0.55) | | 5.5E-29 |
| Model 2^4^ | | 1 | | 0.85 (0.72, 1.02) | 0.79 (0.67, 0.93) | 0.74 (0.62, 0.87) | 0.69 (0.58, 0.82) | 0.69 (0.58, 0.83) | | 0.60 (0.47, 0.76) | | 3.2E-9 |
| Model 3^5^ | | 1 | | 0.85 (0.71, 1.01) | 0.79 (0.67, 0.93) | 0.73 (0.62, 0.86) | 0.69 (0.58, 0.82) | 0.69 (0.58, 0.82) | | 0.61 (0.48, 0.77) | | 3.3E-9 |
| Model 4^6^ | | 1 | | 0.86 (0.72, 1.02) | 0.80 (0.68, 0.94) | 0.75 (0.63, 0.88) | 0.70 (0.59, 0.84) | 0.70 (0.59, 0.84) | | 0.61 (0.48, 0.77) | | 6.9E-9 |
|  | |  | |  |  |  |  |  | |  | |  |
| ^1^Values are HR, 95% CI. Multivariate proportional hazards were used to examine the associations. ^2^P-trend across index categories. ^3^Adjusted for age, sex, dietary assessment version, season, energy intake. ^4^As above plus physical activity, smoking, alcohol use and education. ^5^As above plus BMI. ^6^Adjusted for age, sex, dietary assessment version, season, physical activity, smoking, alcohol use and education. | | | | | | | | | | | | |

Supplemental table 11. Sensitivity analyses of the EAT-Lancet index categories and all-cause mortality in the Malmö Diet and Cancer Study^1^,

|  | | | | | **EAT-Lancet index categories** | | | | | | |  | | | | |
| --- | --- | --- | --- | --- | --- | --- | --- | --- | --- | --- | --- | --- | --- | --- | --- | --- |
| **Diet changers exkl.** | **≤13** | | **14-16** | | | | **17-19** | | **20-22** | | | | **≥23** | | | **P-trend^2^** |
| n/cases | 1,909/691 | | 4,553/1,528 | | | | 6,415/1,976 | | 3,739/1,017 | | | | 1,143/292 | | |  |
| Person years (sum) | 37,055 | | 89,993 | | | | 238,804 | | 76,465 | | | | 23,826 | | |  |
| Model 3^3,4^ | 1 | | 0.90 (0.83, 0.99) | | | | 0.84 (0.77, 0.92) | | 0.78 (0.70, 0.86) | | | | 0.75 (0.65, 0.87) | | | 5.1E-8 |
|  |  | |  | | | |  | |  | | | |  | | |  |
| **Misreporters excluded** | |  | |  | | | |  | | |  | | | |  | |
| n/cases | 1,844/667 | | 4,572/1,578 | | | | 6,653/2,117 | | 3,953/1,211 | | | | 1,363/354 | | |  |
| Person years (sum) | 35,895 | | 90,002 | | | | 133,136 | | 80,526 | | | | 28,341 | | |  |
| Model 3^3,5^ | 1 | | 0.92 (0.84, 1.01) | | | | 0.84 (0.77, 0.92) | | 0.82 (0.74, 0.91) | | | | 0.76 (0.66, 0.87) | | | 7.5E-7 |
|  |  | |  | | | |  | |  | | | |  | | |  |
| **Died < 2 years after baseline excluded** | | | | | |  | | | |  | | | |  | | |
| n/cases | 2,183/796 | | 5,333/1788 | | | | 7,925/2,443 | | 5,011/1,419 | | | | 1,817/452 | | |  |
| Person years (sum) | 42,459 | | 105,919 | | | | 159,952 | | 102,803 | | | | 38,110 | | |  |
| Model 3^3,6^ | 1 | | 0.89 (0.81, 0.96) | | | | 0.83 (0.76, 0.90) | | 0.78 (0.71, 0.86) | | | | 0.73 (0.65, 0.83) | | | 2.3E-9 |
|  |  | |  | | | |  | |  | | | |  | | |  |
| ^1^Values are HR, 95% CI. Multivariate proportional hazards were used to examine the associations. ^2^P-trend across index categories. ^3^Adjusted for: age, sex, dietary assessment version, season, energy intake, physical activity, smoking, alcohol, education, and BMI. ^4^17,759 participants were included in the analysis (4,643 reported change in dietary habits). ^5^18,399 participants were included in the analysis (4,022 are classified as under- or over reporters). ^6^22,269 participants were included in the analysis (152 participants died within two years after baseline). | | | | | | | | | | | | | | | | |

Supplemental table 12. Associations between EAT-Lancet index components (14 food groups) and all-cause mortality in 22,388 participants from the Malmö Diet and Cancer Study stratified according to their score^1^.

|  | EAT-Lancet index component points | | | |  |
| --- | --- | --- | --- | --- | --- |
|  | 0 | 1 | 2 | 3 | p-trend^2^ |
| Whole grains (n) | <58 g (11,034) | 58-116 g (7,282) | 116-232 g (3,521) | >232 g (584) |  |
| Model 1^3^ | 1 | 0.84 (0.80, 0.89) | 0.83 (0.78, 0.89) | 0.72 (0.62, 0.84) | <0.01 |
| Model 2^4^ | 1 | 0.91 (0.86, 0.96) | 0.92 (0.85, 0.98) | 0.81 (0.69, 0.94) | <0.01 |
| Model 4^5^ | 1 | 0.91 (0.86, 0.96) | 0.92 (0.86, 0.99) | 0.82 (0.70, 0.95) | <0.01 |
| Model 5^6^ |  | 0.93 (0.88, 0.98) | 0.95 (0.88, 1.02) | 0.84 (0.72, 0.98) | <0.01 |
| Potatoes (n) | >200 g (2,671) | 100-200 g (10,032) | 50-100 g (7,069) | <50 g (2,649) |  |
| Model 1^3^ | 1 | 0.88 (0.82, 0.95) | 0.85 (0.78, 0.92) | 0.84 (0.75, 0.93) | <0.01 |
| Model 2^4^ | 1 | 0.92 (0.86, 0.99) | 0.91 (0.83, 0.98) | 0.90 (0.81, 1.00) | 0.05 |
| Model 4^5^ | 1 | 0.93 (0.86, 0.99) | 0.91 (0.84, 0.99) | 0.91 (0.82, 1.01) | 0.06 |
| Model 5^6^ |  | 0.93 (0.86, 1.00) | 0.91 (0.84, 0.99) | 0.91 (0.82, 1.02) | 0.15 |
| Vegetables (n) | <100 g (5,824) | 100-200 g (10,456) | 200-300 g (4,403) | >300 g (1,738) |  |
| Model 1^3^ | 1 | 0.85 (0.81, 0.90) | 0.76 (0.71, 0.82) | 0.67 (0.60, 0.74) | <0.01 |
| Model 2^4^ | 1 | 0.91 (0.86, 0.96) | 0.85 (0.79, 0.92) | 0.77 (0.69, 0.86) | <0.01 |
| Model 4^5^ | 1 | 0.91 (0.86, 0.96) | 0.85 (0.79, 0.92) | 0.77 (0.69, 0.86) | <0.01 |
| Model 5^6^ |  | 0.92 (0.87, 0.97) | 0.86 (0.80, 0.93) | 0.78 (0.70, 0.88) | <0.01 |
| Fruits (n) | <50 g (1,942) | 50-100 g (3,275) | 100-200 g (8,273) | >200 g (8,931) |  |
| Model 1^3^ | 1 | 0.85 (0.81, 0.90) | 0.76 (0.71, 0.82) | 0.67 (0.60, 0.74) | <0.01 |
| Model 2^4^ | 1 | 0.91 (0.86, 0.96) | 0.85 (0.79, 0.92) | 0.77 (0.69, 0.86) | <0.01 |
| Model 4^5^ | 1 | 0.91 (0.86, 0.96) | 0.85 (0.79, 0.92) | 0.77 (0.69, 0.86) | <0.01 |
| Model 5^6^ |  | 0.89 (0.81, 0.98) | 0.89 (0.82, 0.98) | 0.87 (0.79, 0.95) | <0.01 |
| Dairy (n) | >1000 g (3,760) | 500-1000 g (12,158) | 250-500 g (5,297) | <250 g (1,206) |  |
| Model 1^3^ | 1 | 0.88 (0.82, 0.94) | 0.88 (0.81, 0.96) | 1.00 (0.89, 1.13) | 0.45 |
| Model 2^4^ | 1 | 0.93 (0.87, 1.00) | 0.94 (0.86, 1.02) | 1.03 (0.91, 1.17) | 0.92 |
| Model 4^5^ | 1 | 0.94 (0.88, 1.00) | 0.94 (0.88, 1.02) | 1.04 (0.93, 1.17) | 0.91 |
| Model 5^6^ |  | 0.95 (0.88, 1.02) | 0.95 (0.87, 1.04) | 1.04 (0.92, 1.19) | 0.48 |
| Beef and lamb (n) | >28 g (14,951) | 14-28 g (4,827) | 7-14 g (1,539) | <7 g (1,104) |  |
| Model 1^3^ | 1 | 0.95 (0.90, 1.01) | 0.95 (0.86, 1.04) | 0.90 (0.80, 1.01) | <0.01 |
| Model 2^4^ | 1 | 0.96 (0.90, 1.01) | 0.95 (0.86, 1.04) | 0.91 (0.81, 1.02) | 0.04 |
| Model 4^5^ | 1 | 0.96 (0.90, 1.02) | 0.95 (0.86, 1.04) | 0.91 (0.81, 1.02) | 0.04 |
| Model 5^6^ |  | 0.96 (0.91, 1.02) | 0.95 (0.86, 1.04) | 0.94 (0.83, 1.06) | 0.17 |
| Pork (n) | >28 g (18,513) | 14-28 g (2,557) | 7-14 g (679) | <7 g (672) |  |
| Model 1^3^ | 1 | 0.93 (0.86, 1.01) | 0.89 (0.77, 1.03) | 0.87 (0.74, 1.02) | <0.01 |
| Model 2^4^ | 1 | 0.98 (0.91, 1.06) | 0.92 (0.80, 1.07) | 0.90 (0.77, 1.06) | 0.11 |
| Model 4^5^ | 1 | 0.98 (0.91, 1.06) | 0.93 (0.80, 1.07) | 0.90 (0.77, 1.06) | 0.11 |
| Model 5^6^ |  | 1.00 (0.92, 1.08) | 0.95 (0.82, 1.10) | 0.97 (0.82, 1.16) | 0.94 |
| Poultry (n) | >116 g (89) | 58-116 g (988) | 29-58 g (4,073) | <29 g (17,271) |  |
| Model 1^3^ | 1 | 0.93 (0.86, 1.01) | 0.89 (0.77, 1.03) | 0.87 (0.74, 1.02) | 0.49 |
| Model 2^4^ | 1 | 0.98 (0.91, 1.06) | 0.92 (0.80, 1.07) | 0.90 (0.77, 1.06) | 0.80 |
| Model 4^5^ | 1 | 0.98 (0.91, 1.06) | 0.93 (0.80, 1.07) | 0.90 (0.77, 1.06) | 0.80 |
| Model 5^6^ |  | 0.75 (0.50, 1.11) | 0.80 (0.54, 1.17) | 0.79 (0.54, 1.16) | 0.86 |
| Eggs (n) | >50 g (1,882) | 25-50 g (6,338) | 13-25 g (6,571) | <13 g (7,630) |  |
| Model 1^3^ | 1 | 0.85 (0.78, 0.93) | 0.79 (0.72, 0.86) | 0.82 (0.75, 0.90) | <0.01 |
| Model 2^4^ | 1 | 0.92 (0.84, 1.00) | 0.86 (0.79, 0.94) | 0.89 (0.82, 0.97) | 0.02 |
| Model 4^5^ | 1 | 0.92 (0.84, 1.00) | 0.86 (0.79, 0.94) | 0.89 (0.82, 0.97) | 0.02 |
| Model 5^6^ |  | 0.92 (0.84, 1.00) | 0.86 (0.78, 0.94) | 0.89 (0.81, 0.97) | 0.02 |
| Fish (n) | <7 g (2.425) | 7-14 g (1,379) | 14-28 g (3,871) | >28 g (14,746) |  |
| Model 1^3^ | 1 | 1.00 (0.88, 1.13) | 0.97 (0.88, 1.07) | 0.90 (0.83, 0.98) | <0.01 |
| Model 2^4^ | 1 | 1.05 (0.92, 1.19) | 1.02 (0.92, 1.13) | 0.97 (0.89, 1.06) | 0.19 |
| Model 4^5^ | 1 | 1.05 (0.93, 1.19) | 1.02 (0.92, 1.13) | 0.97 (0.90, 1.06) | 0.20 |
| Model 5^6^ |  | 1.06 (0.93, 1.20) | 1.03 (0.93, 1.14) | 1.00 (0.92, 1.09) | 0.36 |
| Legumes (n) | <18.75 g (20.311) | 18.75-37.5 g (1,805) | 37.5-75 g (283) | >75 g (22) |  |
| Model 1^3^ | 1 | 1.06 (0.97, 1.15) | 1.24 (1.03, 1.49) | 1.59 (0.8, 3.19) | <0.01 |
| Model 2^4^ | 1 | 1.02 (0.94, 1.11) | 1.24 (1.03, 1.50) | 1.25 (0.63, 2.51) | 0.06 |
| Model 4^5^ | 1 | 1.02 (0.94, 1.11) | 1.24 (1.03, 1.49) | 1.25 (0.62, 2.51) | 0.06 |
| Model 5^6^ |  | 1.03 (0.95, 1.12) | 1.28 (1.06, 1.55) | 1.34 (0.67, 2.70) | 0.04 |
| Nuts (n) | <12.5 g (21.745) | 12.5-25 g (487) | 25-50 g (162) | >50 g (27) |  |
| Model 1^3^ | 1 | 0.92 (0.77, 1.09) | 0.87 (0.65, 1.16) | 0.95 (0.45, 1.99) | 0.20 |
| Model 2^4^ | 1 | 0.96 (0.81, 1.14) | 0.90 (0.67, 1.20) | 0.83 (0.39, 1.73) | 0.33 |
| Model 4^5^ | 1 | 0.96 (0.81, 1.14) | 0.90 (0.67, 1.20) | 0.83 (0.39, 1.73) | 0.33 |
| Model 5^6^ |  | 0.98 (0.82, 1.17) | 0.93 (0.69, 1.24) | 0.90 (0.43, 1.89) | 0.52 |
| Unsaturated oils (n) | <10 g (4.922) | 10-20 g (4,343) | 20-40 g (6,938) | >50 g (6,218) |  |
| Model 1^3^ | 1 | 0.95 (0.89, 1.03) | 0.89 (0.83, 0.95) | 0.95 (0.89, 1.01) | 0.04 |
| Model 2^4^ | 1 | 0.99 (0.92, 1.07) | 0.94 (0.88, 1.00) | 0.98 (0.92, 1.05) | 0.30 |
| Model 4^5^ | 1 | 0.99 (0.92, 1.07) | 0.94 (0.88, 1.00) | 0.98 (0.92, 1.05) | 0.30 |
| Model 5^6^ |  | 0.99 (0.92, 1.07) | 0.95 (0.88, 1.01) | 0.99 (0.92, 1.06) | 0.20 |
| Added sugar (n) | >124 g (880) | 62-124 g (6,971) | 31-62 g (10,047) | <31 g (4,523) |  |
| Model 1^3^ | 1 | 0.85 (0.75, 0.96) | 0.84 (0.74, 0.95) | 0.96 (0.83, 1.10) | 0.16 |
| Model 2^4^ | 1 | 0.90 (0.80, 1.02) | 0.91 (0.80, 1.04) | 0.99 (0.86, 1.14) | 0.18 |
| Model 4^5^ | 1 | 0.90 (0.80, 1.01) | 0.90 (0.80, 1.01) | 0.97 (0.86, 1.10) | 0.29 |
| Model 5^6^ | 1 | 0.91 (0.81, 1.03) | 0.92 (0.81, 1.05) | 0.99 (0.85, 1.15) | 0.08 |
| ^1^Values are given as hazard ratios (HR) and 95% confidence intervals within parentheses with the respective 0 point group as the reference group. g = gram. n = number of participants per index group. Multivariate proportional hazards were used to examine the associations. ^2^P-trend across index categories. ^3^Adjusted for age, sex, dietary assessment version, season, energy intake. ^4^As above plus physical activity, smoking, alcohol use and education. ^5^Adjusted for age, sex, dietary assessment version, season, physical activity, smoking habits, alcohol consumption and education level. ^6^Adjusted as model 3 plus all 14 food groups (whole grains, potatoes, vegetables, fruits, dairy, beef and lamb, pork, poultry, eggs, fish, legumes, nuts, unsaturated oils and added sugar). | | | | | |

Supplemental table 13. Associations between EAT-Lancet index components (14 food groups) and cancer mortality in 22,388 participants from the Malmö Diet and Cancer Study stratified according to their score^1^.

|  | EAT-Lancet index component points | | | | | | |  | | |  |
| --- | --- | --- | --- | --- | --- | --- | --- | --- | --- | --- | --- |
|  | 0 | 1 | | 2 | | 3 | | | p-trend^2^ | |  |
| Whole grains (n) | <58 g (11,015) | 58-116 g (7,273) | | 116-232 g (3,517) | | >232 g (583) | | |  | |  |
| Model 1^3^ | 1 | 0.89 (0.81, 0.97) | | 0.87 (0.78, 0.97) | | 0.65 (0.49, 0.84) | | | <0.01 | |  |
| Model 2^4^ | 1 | 0.96 (0.88, 1.04) | | 0.96 (0.86, 1.07) | | 0.72 (0.55, 0.95) | | | 0.07 | |  |
| Model 3^5^ | 1 | 0.96 (0.88, 1.04) | | 0.96 (0.86, 1.08) | | 0.73 (0.56, 0.95) | | | 0.08 | |  |
| Model 4^6^ | 1 | 0.96 (0.88, 1.04) | | 0.96 (0.86, 1.07) | | 0.72 (0.56, 0.94) | | | 0.06 | |  |
| Potatoes (n) | >200 g (2,669) | 100-200 g (10,014) | | 50-100 g (7,061) | | <50 g (2,644) | | |  | |  |
| Model 1^3^ | 1 | 0.87 (0.77, 0.98) | | 0.84 (0.74, 0.96) | | 0.79 (0.67, 0.94) | | | 0.01 | |  |
| Model 2^4^ | 1 | 0.90 (0.80, 1.02) | | 0.90 (0.78, 1.02) | | 0.85 (0.72, 1.01) | | | 0.10 | |  |
| Model 3^5^ | 1 | 0.90 (0.80, 1.02) | | 0.90 (0.78, 1.02) | | 0.85 (0.72, 1.01) | | | 0.10 | |  |
| Model 4^6^ | 1 | 0.91 (0.81, 1.03) | | 0.91 (0.80, 1.04) | | 0.87 (0.74, 1.03) | | | 0.15 | |  |
| Vegetables (n) | <100 g (5,815) | 100-200 g (10,443) | | 200-300 g (4,395) | | >300 g (1,735) | | |  | |  |
| Model 1^3^ | 1 | 0.90 (0.82, 0.99) | | 0.80 (0.71, 0.9) | | 0.62 (0.51, 0.74) | | | <0.01 | |  |
| Model 2^4^ | 1 | 0.96 (0.87, 1.05) | | 0.90 (0.80, 1.01) | | 0.70 (0.58, 0.85) | | | <0.01 | |  |
| Model 3^5^ | 1 | 0.95 (0.87, 1.04) | | 0.89 (0.79, 1.01) | | 0.70 (0.58, 0.84) | | | <0.01 | |  |
| Model 4^6^ | 1 | 0.96 (0.87, 1.05) | | 0.90 (0.80, 1.01) | | 0.70 (0.58, 0.84) | | | <0.01 | |  |
| Fruit (n) | <50 g (1,938) | 50-100 g (3,271) | | 100-200 g (8,266) | | >200 g (8,913) | | |  | |  |
| Model 1^3^ | 1 | 0.86 (0.74, 1.00) | | 0.77 (0.67, 0.89) | | 0.69 (0.60, 0.79) | | | <0.01 | |  |
| Model 2^4^ | 1 | 0.94 (0.80, 1.09) | | 0.93 (0.81, 1.06) | | 0.88 (0.76, 1.02) | | | 0.07 | |  |
| Model 3^5^ | 1 | 0.94 (0.80, 1.09) | | 0.92 (0.80, 1.06) | | 0.87 (0.75, 1.01) | | | 0.05 | |  |
| Model 4^6^ | 1 | 0.94 (0.80, 1.09) | | 0.92 (0.80, 1.06) | | 0.88 (0.76, 1.01) | | | 0.06 | |  |
| Dairy (n) | >1000 g (3,755) | 500-1000 g (12,141) | | 250-500 g (5,288) | | <250 g (1,204) | | |  | |  |
| Model 1^3^ | 1 | 0.87 (0.78, 0.97) | | 0.85 (0.74, 0.98) | | 0.93 (0.76, 1.14) | | | 0.19 | |  |
| Model 2^4^ | 1 | 0.92 (0.82, 1.03) | | 0.90 (0.78, 1.03) | | 0.97 (0.79, 1.19) | | | 0.41 | |  |
| Model 3^5^ | 1 | 0.92 (0.83, 1.03) | | 0.90 (0.79, 1.04) | | 0.97 (0.79, 1.19) | | | 0.44 | |  |
| Model 4^6^ | 1 | 0.95 (0.85, 1.05) | | 0.93 (0.83, 1.05) | | 1.01 (0.84, 1.22) | | | 0.64 | |  |
| Beef and lamb (n) | >28 g (14,928) | 14-28 g (4,820) | | 7-14 g (1,537) | | <7 g (1,103) | | |  | |  |
| Model 1^3^ | 1 | 0.91 (0.83, 1.01) | | 0.96 (0.82, 1.12) | | 0.88 (0.72, 1.06) | | | 0.61 | |  |
| Model 2^4^ | 1 | 0.92 (0.83, 1.02) | | 0.96 (0.82, 1.12) | | 0.89 (0.74, 1.08) | | | 0.12 | |  |
| Model 3^5^ | 1 | 0.92 (0.84, 1.02) | | 0.96 (0.82, 1.12) | | 0.90 (0.74, 1.09) | | | 0.14 | |  |
| Model 4^6^ | 1 | 0.93 (0.84, 1.02) | | 0.96 (0.83, 1.12) | | 0.90 (0.74, 1.09) | | | 0.14 | |  |
| Pork (n) | >28 g (18,485) | 14-28 g (2,553) | | 7-14 g (679) | | <7 g (671) | | |  | |  |
| Model 1^3^ | 1 | 0.91 (0.80, 1.03) | | 0.88 (0.70, 1.12) | | 0.74 (0.57, 0.97) | | | 0.01 | |  |
| Model 2^4^ | 1 | 0.96 (0.84, 1.09) | | 0.93 (0.73, 1.18) | | 0.78 (0.59, 1.02) | | | 0.06 | |  |
| Model 3^5^ | 1 | 0.96 (0.84, 1.09) | | 0.93 (0.74, 1.18) | | 0.78 (0.59, 1.02) | | | 0.18 | |  |
| Model 4^6^ | 1 | 0.96 (0.85, 1.09) | | 0.93 (0.74, 1.18) | | 0.78 (0.59, 1.02) | | | 0.07 | |  |
| Poultry (n) | >116 g (89) | 58-116 g (983) | | 29-58 g (4,066) | | <29 g (17,250) | | |  | |  |
| Model 1^3^ | 1 | 1.04 (0.53, 2.05) | | 1.03 (0.53, 2.00) | | 1.03 (0.53, 1.98) | | | 0.92 | |  |
| Model 2^4^ | 1 | 1.02 (0.52, 2.01) | | 1.02 (0.52, 1.97) | | 1.00 (0.52, 1.92) | | | 0.68 | |  |
| Model 3^5^ | 1 | 1.03 (0.52, 2.03) | | 1.03 (0.53, 1.99) | | 1.01 (0.52, 1.95) | | | 0.76 | |  |
| Model 4^6^ | 1 | 1.02 (0.52, 2.02) | | 1.02 (0.53, 1.97) | | 1.00 (0.52, 1.93) | | | 0.70 | |  |
| Eggs (n) | >50 g (1,881) | 25-50 g (6,329) | | 13-25 g (6,558) | | <13 g (7,620) | | |  | |  |
| Model 1^3^ | 1 | 0.89 (0.77, 1.02) | | 0.77 (0.67, 0.88) | | 0.81 (0.70, 0.93) | | | 0.00 | |  |
| Model 2^4^ | 1 | 0.95 (0.83, 1.09) | | 0.84 (0.72, 0.96) | | 0.88 (0.76, 1.01) | | | 0.03 | |  |
| Model 3^5^ | 1 | 0.95 (0.83, 1.09) | | 0.84 (0.73, 0.97) | | 0.89 (0.77, 1.02) | | | 0.03 | |  |
| Model 4^6^ | 1 | 0.95 (0.83, 1.10) | | 0.84 (0.73, 0.97) | | 0.89 (0.77, 1.02) | | | 0.04 | |  |
| Fish (n) | <7 g (2,419) | 7-14 g (1,377) | | 14-28 g (3,867) | | >28 g (14,725) | | |  | |  |
| Model 1^3^ | 1 | 1.17 (0.96, 1.43) | | 1.09 (0.93, 1.28) | | 1.05 (0.91, 1.20) | | | 0.87 | |  |
| Model 2^4^ | 1 | 1.22 (1.00, 1.50) | | 1.14 (0.97, 1.33) | | 1.12 (0.98, 1.29) | | | 0.36 | |  |
| Model 3^5^ | 1 | 1.22 (1.00, 1.49) | | 1.13 (0.96, 1.33) | | 1.12 (0.97, 1.29) | | | 0.39 | |  |
| Model 4^6^ | 1 | 1.22 (1.00, 1.49) | | 1.13 (0.97, 1.33) | | 1.12 (0.97, 1.29) | | | 0.38 | |  |
| Legumes (n) | <18.75 g (20,282) | 18.75-37.5 g (1,802) | | 37.5-75 g (282) | | >75 g (22) | | |  | |  |
| Model 1^3^ | 1 | 0.96 (0.84, 1.10) | | 1.31 (0.98, 1.76) | | 1.46 (0.47, 4.52) | | | 0.42 | |  |
| Model 2^4^ | 1 | 0.93 (0.81, 1.07) | | 1.31 (0.98, 1.76) | | 1.33 (0.43, 4.13) | | | 0.69 | |  |
| Model 3^5^ | 1 | 0.93 (0.81, 1.07) | | 1.31 (0.98, 1.76) | | 1.36 (0.44, 4.24) | | | 0.67 | |  |
| Model 4^6^ | 1 | 0.93 (0.81, 1.06) | | 1.30 (0.97,1.75) | | 1.32 (0.42-4.09) | | | 0.73 | |  |
| Nuts (n) | <12.5 g (21,713) | 12.5-25 g (486) | | 25-50 g (162) | | >50 g (27) | | |  | |  |
| Model 1^3^ | 1 | 1 (0.76, 1.30) | | 0.94 (0.60, 1.48) | | 1.05 (0.34, 3.26) | | | 0.87 | |  |
| Model 2^4^ | 1 | 1.04 (0.80, 1.36) | | 0.97 (0.62, 1.52) | | 0.95 (0.31, 2.95) | | | 0.97 | |  |
| Model 3^5^ | 1 | 1.05 (0.80, 1.36) | | 0.97 (0.62, 1.53) | | 0.96 (0.31, 2.99) | | | 0.94 | |  |
| Model 4^6^ | 1 | 1.04 (0.80, 1.36) | | 0.96 (0.61, 1.51) | | 0.94 (0.30, 2.93) | | | 0.99 | |  |
| Unsaturated oils (n) | <10 g (4,910) | 10-20 g (4,338) | | 20-40 g (6,933) | | >50 g (6,207) | | |  | |  |
| Model 1^3^ | 1 | 0.96 (0.85, 1.08) | | 0.88 (0.79, 0.98) | | 0.93 (0.83, 1.04) | | | 0.10 | |  |
| Model 2^4^ | 1 | 1.00 (0.88, 1.12) | | 0.93 (0.84, 1.04) | | 0.96 (0.86, 1.07) | | | 0.28 | |  |
| Model 3^5^ | 1 | 0.99 (0.88, 1.12) | | 0.93 (0.83, 1.03) | | 0.96 (0.86, 1.07) | | | 0.26 | |  |
| Model 4^6^ | 1 | 1.00 (0.88, 1.12) | | 0.93 (0.84, 1.04) | | 0.95 (0.86, 1.06) | | | 0.25 | |  |
| Added sugar (n) | >124 g (878) | | 62-124 g (6,960) | | 31-62 g (10,034) | | <31 g (4,516) | | |  | |
| Model 1^3^ | 1 | 0.93 (0.76, 1.14) | | 0.91 (0.74, 1.13) | | 1.00 (0.79, 1.26) | | | 0.60 | |  |
| Model 2^4^ | 1 | 1.00 (0.82, 1.22) | | 1.01 (0.81, 1.25) | | 1.05 (0.83, 1.33) | | | 0.54 | |  |
| Model 3^5^ | 1 | 1.00 (0.81, 1.22) | | 1.00 (0.81, 1.24) | | 1.03 (0.82, 1.31) | | | 0.65 | |  |
| Model 4^6^ | 1 | 1.00 (0.82, 1.22) | | 1.01 (0.83, 1.23) | | 1.05 (0.86, 1.30) | | | 0.42 | |  |
| ^1^Values are given as hazard ratios (HR) and 95% confidence intervals within parentheses, with the respective 0 point group as the reference group. g = gram, n = number of participants per index group. Multivariate proportional hazards were used to examine the associations. 33 participants with missing values about death cause were excluded from the total sample (22,388 included). ^2^P-trend across index categories. ^3^Adjusted for age, sex, dietary assessment version, season, energy intake. ^4^As above plus physical activity, smoking, alcohol use and education. ^5^As above plus BMI. ^6^ Adjusted for age, sex, dietary assessment version, season, physical activity, smoking habits, alcohol consumption and education level. | | | | | | | | | | |  |

**Supplemental table 14**. Associations between EAT-Lancet index components (14 food groups) and cardiovascular mortality in 22,388 participants from the Malmö Diet and Cancer Study stratified according to their score^1^.

|  | EAT-Lancet index component points | | | |  | |
| --- | --- | --- | --- | --- | --- | --- |
|  | 0 | 1 | 2 | 3 | | p-trend^2^ |
| Whole grains (n) | <58 g (11,015) | 58-116 g (7,273) | 116-232 g (3,517) | >232 g (583) | |  |
| Model 1^3^ | 1 | 0.77 (0.70, 0.85) | 0.75 (0.66, 0.84) | 0.74 (0.57, 0.95) | | <0.01 |
| Model 2^4^ | 1 | 0.83 (0.75, 0.91) | 0.82 (0.72, 0.93) | 0.83 (0.64, 1.07) | | <0.01 |
| Model 3^5^ | 1 | 0.83 (0.75, 0.92) | 0.83 (0.73, 0.94) | 0.84 (0.65, 1.09) | | <0.01 |
| Model 4^6^ | 1 | 0.83 (0.76, 0.92) | 0.82 (0.73, 0.93) | 0.84 (0.65, 1.08) | | <0.01 |
| Potatoes (n) | >200 g (2,669) | 100-200 g (10,014) | 50-100 g (7,061) | <50 g (2,644) | |  |
| Model 1^3^ | 1 | 0.90 (0.79, 1.02) | 0.88 (0.76, 1.02) | 0.86 (0.71, 1.04) | | 0.13 |
| Model 2^4^ | 1 | 0.94 (0.83, 1.07) | 0.95 (0.82, 1.1) | 0.94 (0.77, 1.14) | | 0.64 |
| Model 3^5^ | 1 | 0.95 (0.83, 1.08) | 0.95 (0.82, 1.1) | 0.95 (0.78, 1.15) | | 0.65 |
| Model 4^6^ | 1 | 0.95 (0.83, 1.08) | 0.96 (0.83, 1.1) | 0.95 (0.79, 1.14) | | 0.69 |
| Vegetables (n) | <100 g (5,815) | 100-200 g (10,443) | 200-300 g (4,395) | >300 g (1,735) | |  |
| Model 1^3^ | 1 | 0.82 (0.75, 0.91) | 0.76 (0.66, 0.86) | 0.72 (0.60, 0.88) | | <0.01 |
| Model 2^4^ | 1 | 0.88 (0.80, 0.97) | 0.85 (0.75, 0.98) | 0.85 (0.70, 1.03) | | 0.01 |
| Model 3^5^ | 1 | 0.87 (0.79, 0.96) | 0.84 (0.73, 0.96) | 0.83 (0.69, 1.01) | | <0.01 |
| Model 4^6^ | 1 | 0.88 (0.80, 0.97) | 0.86 (0.75, 0.98) | 0.85 (0.70, 1.03) | | 0.01 |
| Fruit (n) | <50 g (1,938) | 50-100 g (3,271) | 100-200 g (8,266) | >200 g (8,913) | |  |
| Model 1^3^ | 1 | 0.73 (0.62, 0.86) | 0.67 (0.58, 0.78) | 0.58 (0.50, 0.68) | | <0.01 |
| Model 2^4^ | 1 | 0.79 (0.67, 0.94) | 0.80 (0.69, 0.93) | 0.74 (0.63, 0.86) | | <0.01 |
| Model 3^5^ | 1 | 0.79 (0.67, 0.94) | 0.79 (0.68, 0.92) | 0.72 (0.62, 0.84) | | <0.01 |
| Model 4^6^ | 1 | 0.79 (0.67, 0.94) | 0.80 (0.69, 0.93) | 0.74 (0.64, 0.87) | | <0.01 |
| Dairy (n) | >1000 g (3,755) | 500-1000 g (12,141) | 250-500 g (5,288) | <250 g (1,204) | |  |
| Model 1^3^ | 1 | 0.85 (0.75, 0.96) | 0.86 (0.74, 1.00) | 1.13 (0.92, 1.40) | | 0.78 |
| Model 2^4^ | 1 | 0.91 (0.80, 1.03) | 0.91 (0.78, 1.07) | 1.16 (0.94, 1.44) | | 0.50 |
| Model 3^5^ | 1 | 0.92 (0.81, 1.04) | 0.93 (0.80, 1.08) | 1.19 (0.96, 1.47) | | 0.36 |
| Model 4^6^ | 1 | 0.92 (0.82, 1.03) | 0.92 (0.81, 1.06) | 1.18 (0.97, 1.43) | | 0.50 |
| Beef and lamb (n) | >28 g (14,928) | 14-28 g (4,820) | 7-14 g (1,537) | <7 g (1,103) | |  |
| Model 1^3^ | 1 | 0.98 (0.89, 1.09) | 0.85 (0.71, 1.02) | 0.85 (0.68, 1.05) | | 0.03 |
| Model 2^4^ | 1 | 0.99 (0.89, 1.10) | 0.85 (0.72, 1.02) | 0.86 (0.69, 1.07) | | 0.06 |
| Model 3^5^ | 1 | 0.99 (0.90, 1.10) | 0.86 (0.72, 1.03) | 0.89 (0.72, 1.11) | | 0.12 |
| Model 4^6^ | 1 | 0.99 (0.89, 1.10) | 0.86 (0.72, 1.02) | 0.86 (0.70, 1.07) | | 0.07 |
| Pork (n) | >28 g (18,485) | 14-28 g (2,553) | 7-14 g (679) | <7 g (671) | |  |
| Model 1^3^ | 1 | 0.95 (0.82, 1.09) | 0.87 (0.67, 1.13) | 0.84 (0.62, 1.13) | | 0.10 |
| Model 2^4^ | 1 | 1.01 (0.88, 1.17) | 0.91 (0.70, 1.19) | 0.87 (0.64, 1.19) | | 0.38 |
| Model 3^5^ | 1 | 1.03 (0.89, 1.18) | 0.93 (0.71, 1.20) | 0.92 (0.67, 1.24) | | 0.88 |
| Model 4^6^ | 1 | 1.01 (0.88, 1.17) | 0.92 (0.71, 1.19) | 0.88 (0.65, 1.19) | | 0.40 |
| Poultry (n) | >116 g (89) | 58-116 g (983) | 29-58 g (4,066) | <29 g (17,250) | |  |
| Model 1^3^ | 1 | 0.43 (0.24, 0.79) | 0.48 (0.27, 0.86) | 0.49 (0.28, 0.87) | | 0.74 |
| Model 2^4^ | 1 | 0.44 (0.24, 0.81) | 0.50 (0.28, 0.89) | 0.50 (0.28, 0.88) | | 0.96 |
| Model 3^5^ | 1 | 0.47 (0.26, 0.86) | 0.53 (0.30, 0.94) | 0.54 (0.30, 0.95) | | 0.66 |
| Model 4^6^ | 1 | 0.44 (0.24, 0.81) | 0.5 (0.28, 0.89) | 0.5 (0.28, 0.88) | | 0.95 |
| Eggs (n) | >50 g (1,881) | 25-50 g (6,329) | 13-25 g (6,558) | <13 g (7,620) | |  |
| Model 1^3^ | 1 | 0.82 (0.70, 0.96) | 0.79 (0.68, 0.92) | 0.82 (0.70, 0.95) | | 0.08 |
| Model 2^4^ | 1 | 0.88 (0.75, 1.03) | 0.85 (0.73, 1.00) | 0.88 (0.75, 1.03) | | 0.26 |
| Model 3^5^ | 1 | 0.89 (0.76, 1.04) | 0.87 (0.74, 1.01) | 0.90 (0.77, 1.05) | | 0.44 |
| Model 4^6^ | 1 | 0.88 (0.76, 1.03) | 0.86 (0.73, 1.00) | 0.88 (0.76, 1.03) | | 0.29 |
| Fish (n) | <7 g (2,419) | 7-14 g (1,377) | 14-28 g (3,867) | >28 g (14,725) | |  |
| Model 1^3^ | 1 | 0.91 (0.73, 1.15) | 0.88 (0.74, 1.05) | 0.82 (0.70, 0.95) | | <0.01 |
| Model 2^4^ | 1 | 0.97 (0.77, 1.22) | 0.92 (0.77, 1.10) | 0.89 (0.77, 1.04) | | 0.10 |
| Model 3^5^ | 1 | 0.96 (0.77, 1.21) | 0.91 (0.77, 1.09) | 0.87 (0.75, 1.02) | | <0.05 |
| Model 4^6^ | 1 | 0.97 (0.77, 1.22) | 0.92 (0.77, 1.1) | 0.89 (0.77, 1.04) | | 0.10 |
| Legumes (n) | <18.75 g (20,282) | 18.75-37.5 g (1,802) | 37.5-75 g (282) | >75 g (22) | |  |
| Model 1^3^ | 1 | 1.04 (0.90, 1.20) | 1.14 (0.82, 1.60) | 1.25 (0.31, 4.99) | | 0.36 |
| Model 2^4^ | 1 | 1.00 (0.87, 1.16) | 1.13 (0.81, 1.58) | 0.87 (0.22, 3.50) | | 0.68 |
| Model 3^5^ | 1 | 1.00 (0.87, 1.16) | 1.12 (0.80, 1.56) | 0.95 (0.24, 3.83) | | 0.70 |
| Model 4^6^ |  | 1.00 (0.87, 1.16) | 1.13 (0.81, 1.58) | 0.87 (0.22, 3.49) | | 0.70 |
| Nuts (n) | <12.5 g (21,713) | 12.5-25 g (486) | 25-50 g (162) | >50 g (27) | |  |
| Model 1^3^ | 1 | 0.77 (0.55, 1.07) | 0.89 (0.53, 1.48) | 0.86 (0.21, 3.43) | | 0.19 |
| Model 2^4^ | 1 | 0.81 (0.58, 1.14) | 0.94 (0.57, 1.57) | 0.72 (0.18, 2.89) | | 0.30 |
| Model 3^5^ | 1 | 0.82 (0.59, 1.15) | 0.94 (0.57, 1.57) | 0.79 (0.20, 3.18) | | 0.35 |
| Model 4^6^ | 1 | 0.81 (0.58, 1.14) | 0.94 (0.56, 1.57) | 0.72 (0.18, 2.88) | | 0.30 |
| Unsaturated oils (n) | <10 g (4,910) | 10-20 g (4,338) | 20-40 g (6,933) | >50 g (6,207) | |  |
| Model 1^3^ | 1 | 0.99 (0.86, 1.13) | 0.95 (0.85, 1.07) | 1.00 (0.89, 1.13) | | 0.93 |
| Model 2^4^ | 1 | 1.02 (0.89, 1.17) | 1.00 (0.89, 1.13) | 1.03 (0.92, 1.19) | | 0.68 |
| Model 3^5^ | 1 | 1.02 (0.89, 1.17) | 1.00 (0.88, 1.12) | 1.03 (0.92, 1.17) | | 0.69 |
| Model 4^6^ | 1 | 1.02 (0.89, 1.17) | 1.01 (0.89, 1.13) | 1.03 (0.91, 1.16) | | 0.71 |
| Added sugar (n) | >124 g (878) | 62-124 g (6,960) | 31-62 g (10,034) | <31 g (4,516) | |  |
| Model 1^3^ | 1 | 0.69 (0.57, 0.85) | 0.72 (0.58, 0.89) | 0.84 (0.66, 1.07) | | 0.31 |
| Model 2^4^ | 1 | 0.73 (0.60, 0.90) | 0.77 (0.62, 0.96) | 0.86 (0.67, 1.11) | | 0.34 |
| Model 3^5^ | 1 | 0.72 (0.59, 0.89) | 0.75 (0.61, 0.94) | 0.81 (0.64, 1.04) | | 0.70 |
| Model 4^6^ | 1 | 0.73 (0.60, 0.89) | 0.77 (0.64, 0.94) | 0.86 (0.70, 1.06) | | 0.37 |
| ^1^Values are given as hazard ratios (HR) and 95% confidence intervals within parentheses, with the respective 0 point group as the reference group. g = gram, n = number of participants per index group. Multivariate proportional hazards were used to examine the associations. 33 participants with missing values about death cause were excluded from the total sample (22,388 included). ^2^P-trend across index categories. ^3^Adjusted for age, sex, dietary assessment version, season, energy intake. ^4^As above plus physical activity, smoking, alcohol use and education. ^5^As above plus BMI. ^6^Adjusted for age, sex, dietary assessment version, season, physical activity, smoking habits, alcohol consumption and education level. | | | | | | |

Supplemental figure 1. Flow chart of final sample from the Malmö Diet and Cancer Study.


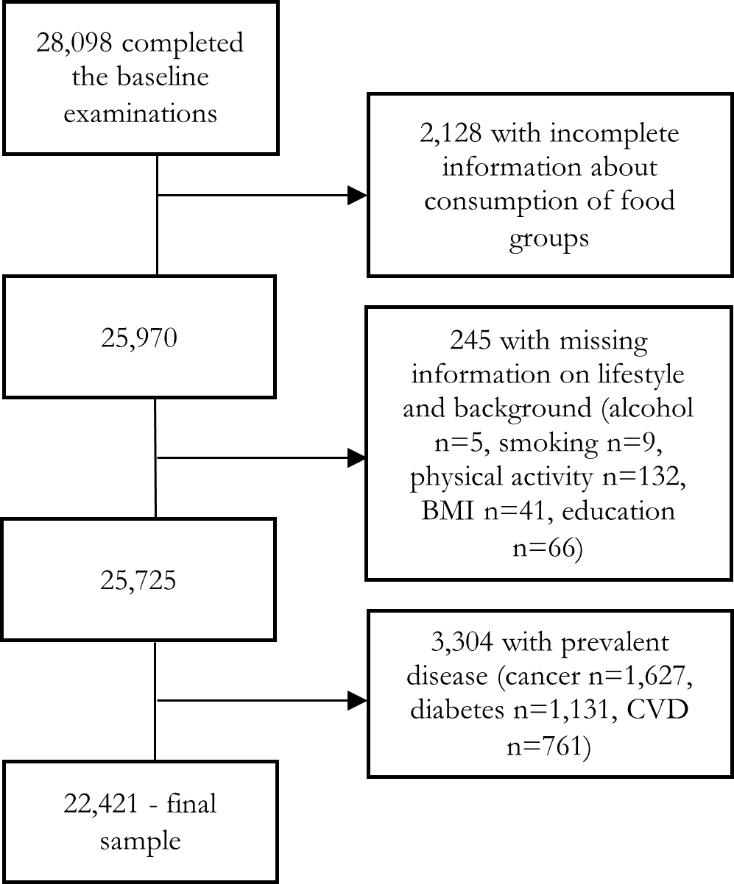


Supplemental figure 2. Restricted cubic splines between EAT-Lancet index and included food components (grams per day) and risk of all-cause mortality using Cox regression, adjusted for age, sex, dietary assessment version, season, energy intake, physical activity, smoking, alcohol use and education and BMI, based on 22, 421 participants from the Malmö Diet and Cancer Study. Solid line is Hazard Ratio and dotted line is 95% CI.
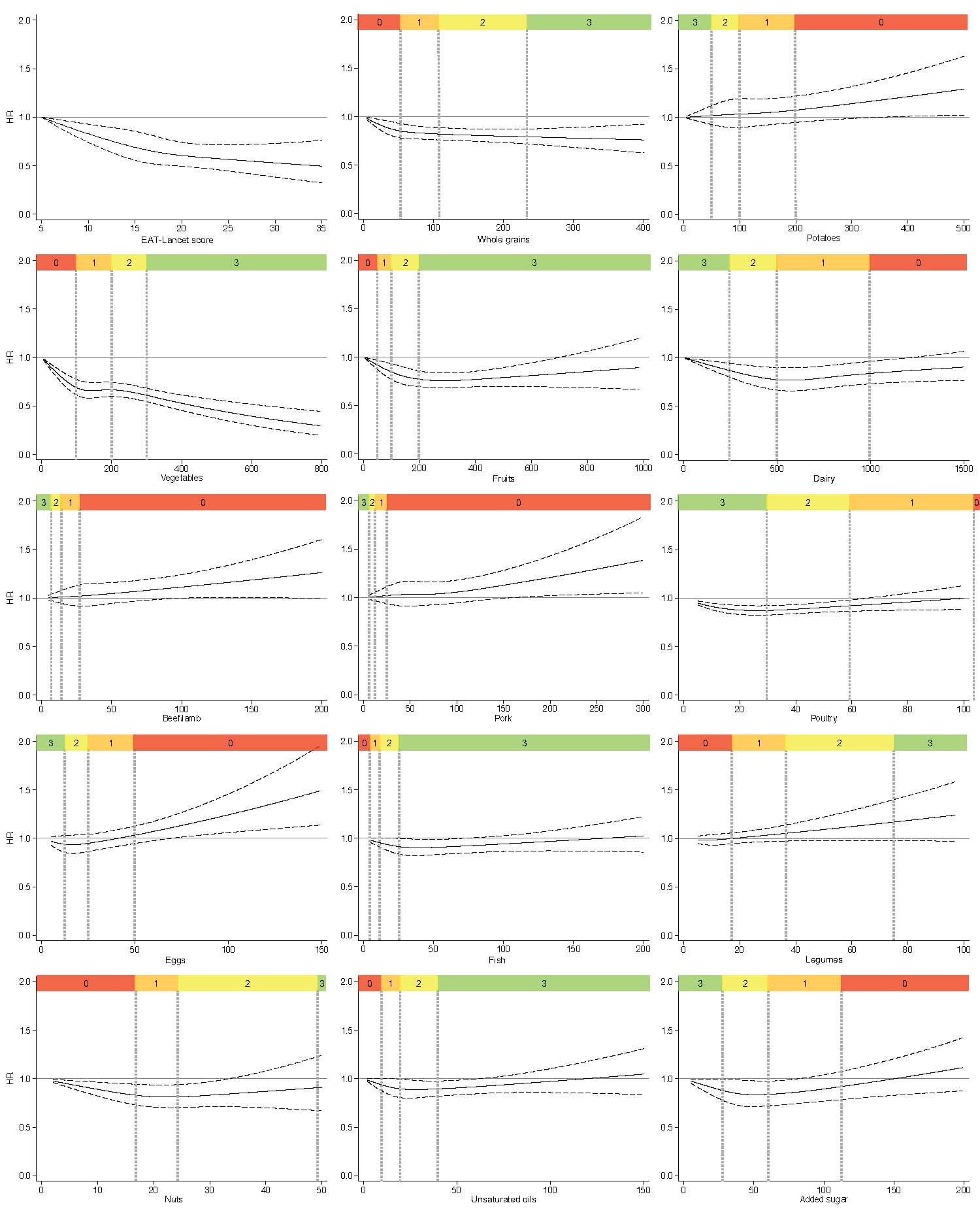


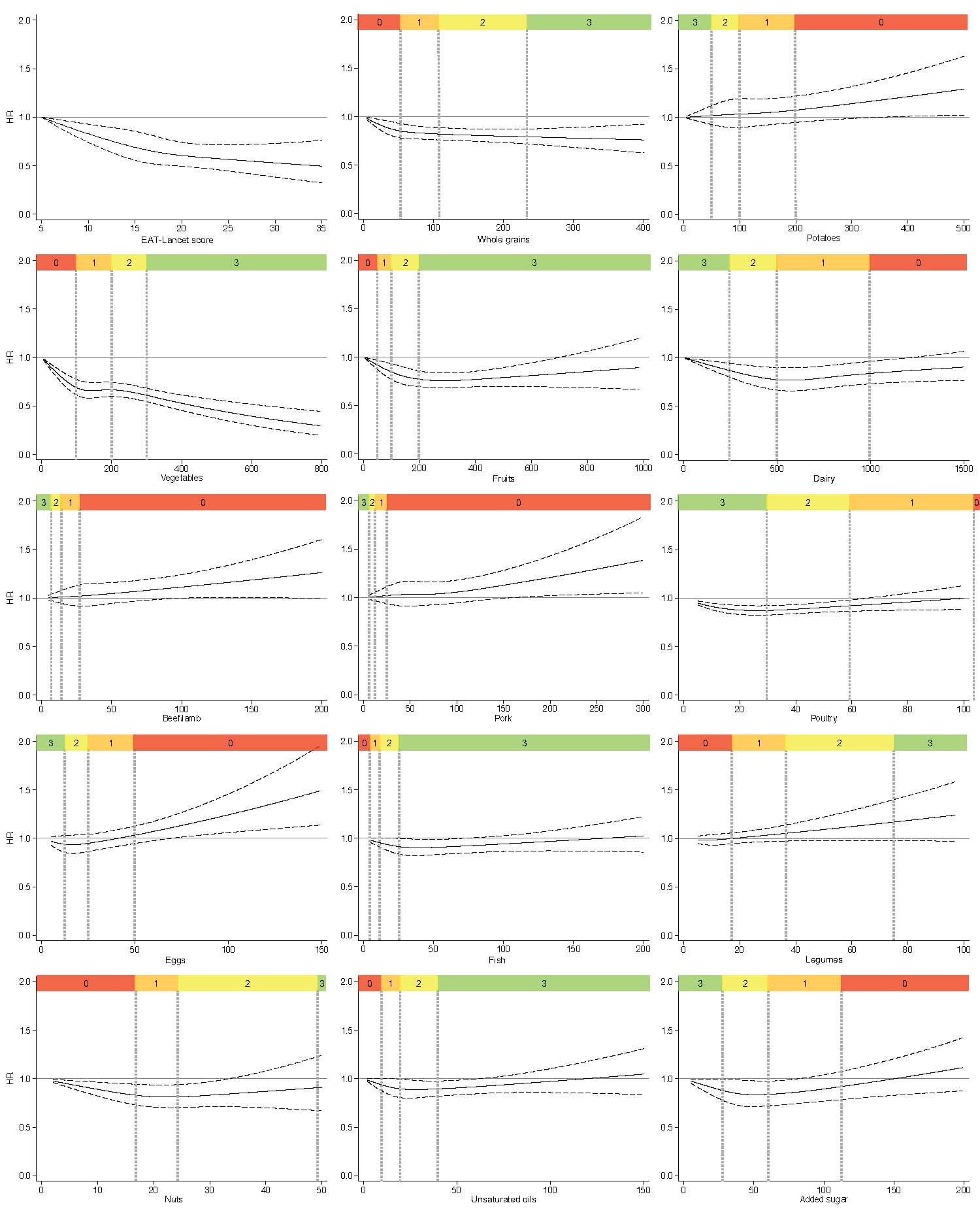


**Supplemental figure 3**. Kaplan-Meier curves showing all-cause mortality for participants stratified by EAT-Lancet index categories, based on 22,421 participants from the Malmö Diet and Cancer Study.


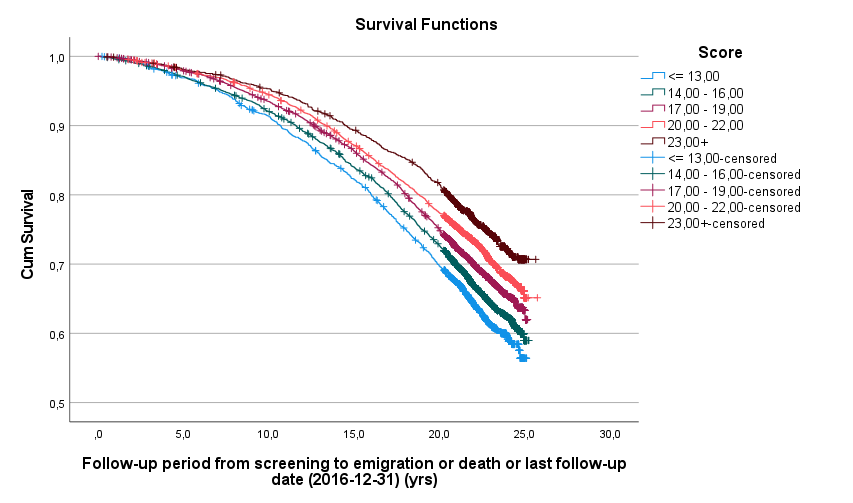


**Supplemental figure 4.** Kaplan-Meier curves showing all-cause mortality for participants stratified by EAT-Lancet index categories, based on 13,853 women from the Malmö Diet and Cancer Study


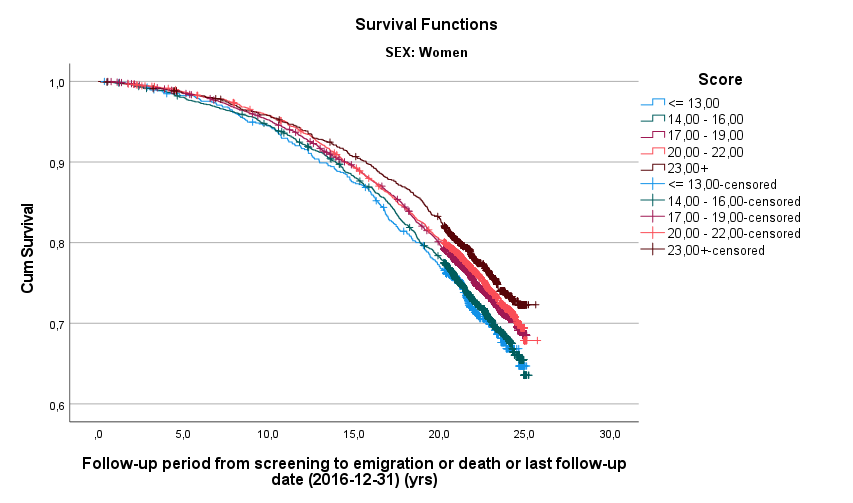


**Supplemental figure 5.** Kaplan-Meier curves showing all-cause mortality for participants stratified by EAT-Lancet index categories, based on 8,568 men from the Malmö Diet and Cancer Study


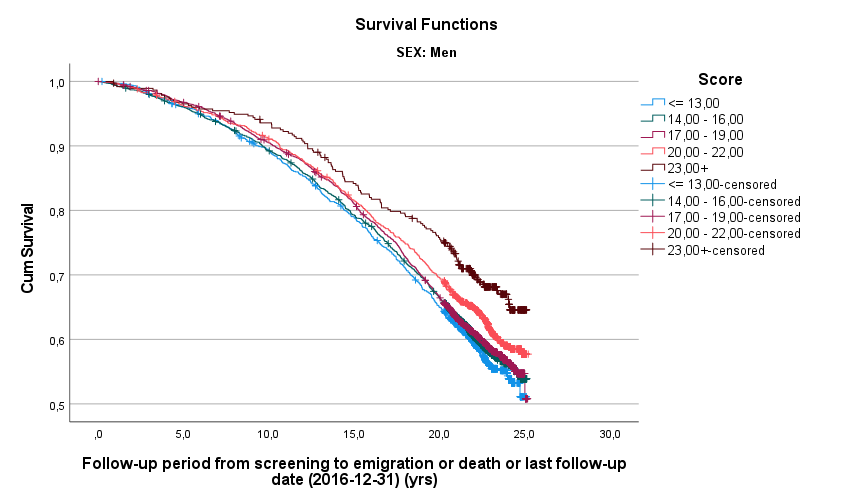


# **References for Supplemental material:**

1. Willett, W., J. Rockstrom, B. Loken, M. Springmann, T. Lang, S. Vermeulen, T. Garnett, D. Tilman, F. DeClerck, A. Wood, et al., *Food in the Anthropocene: the EAT-Lancet Commission on healthy diets from sustainable food systems*. Lancet, 2019. DOI: 10.1016/s0140-6736(18)31788-4.

2. Hellstrand S, O.F., Smith E, Brunkwall L, Ramne S, Sonestedt E, Nilsson P.M, Melander O, Orho-Melander M, Ericson U., *Dietary Data in the Malmö Offspring Study – Reproducibility, Method Comparison and Validation against Objective Biomarkers.* . Nutrients, 2021. 13(5): p. 1579.

3. Stockholm Resilience Centre, *Nordic food systems for improved health and sustainability. Baseline assessment to inform transformation*. 2019.

4. Ramne, S., J. Alves Dias, E. González-Padilla, K. Olsson, B. Lindahl, G. Engström, U. Ericson, I. Johansson, and E. Sonestedt, *Association between added sugar intake and mortality is nonlinear and dependent on sugar source in 2 Swedish population-based prospective cohorts*. Am J Clin Nutr, 2019. 109(2): p. 411-423. DOI: 10.1093/ajcn/nqy268.
